# Preserving a Comprehensive Vegetation Knowledge Base – An Evaluation of Four Historical Soviet Vegetation Maps of the Western Pamirs (Tajikistan)

**DOI:** 10.1371/journal.pone.0148930

**Published:** 2016-02-16

**Authors:** Kim André Vanselow, Cyrus Samimi, Siegmar-W. Breckle

**Affiliations:** 1 Department of Geography, University of Erlangen-Nuremberg, Erlangen, Germany; 2 Department of Geography, University of Bayreuth, Bayreuth, Germany; 3 Department of Ecology, University of Bielefeld, Bielefeld, Germany; 4 BayCEER, University of Bayreuth, Bayreuth, Germany; Trier University, GERMANY

## Abstract

We edited, redrew, and evaluated four unpublished historical vegetation maps of the Western Pamirs (Tajikistan) by the Soviet geobotanist Okmir E. Agakhanjanz. These maps cover an area of 5,188 km^2^ and date from 1958 to 1960. The purpose of this article is to make the historic vegetation data available to the scientific community and thus preserve a hitherto non available and up to now neglected or forgotten data source with great potential for studies on vegetation and ecosystem response to global change. The original hand-drawn maps were scanned, georeferenced, and digitized and the corresponding land cover class was assigned to each polygon. The partly differing legends were harmonized and plant names updated. Furthermore, a digital elevation model and generalized additive models were used to calculate response curves of the land cover classes and to explore vegetation-topography relationships quantitatively. In total, 2,216 polygons belonging to 13 major land cover classes were included that are characterized by 252 different plant species. As such, the presented maps provide excellent comparison data for studies on vegetation and ecosystem change in an area that is deemed to be an important water tower in Central Asia.

## Introduction

**Fig 1:**
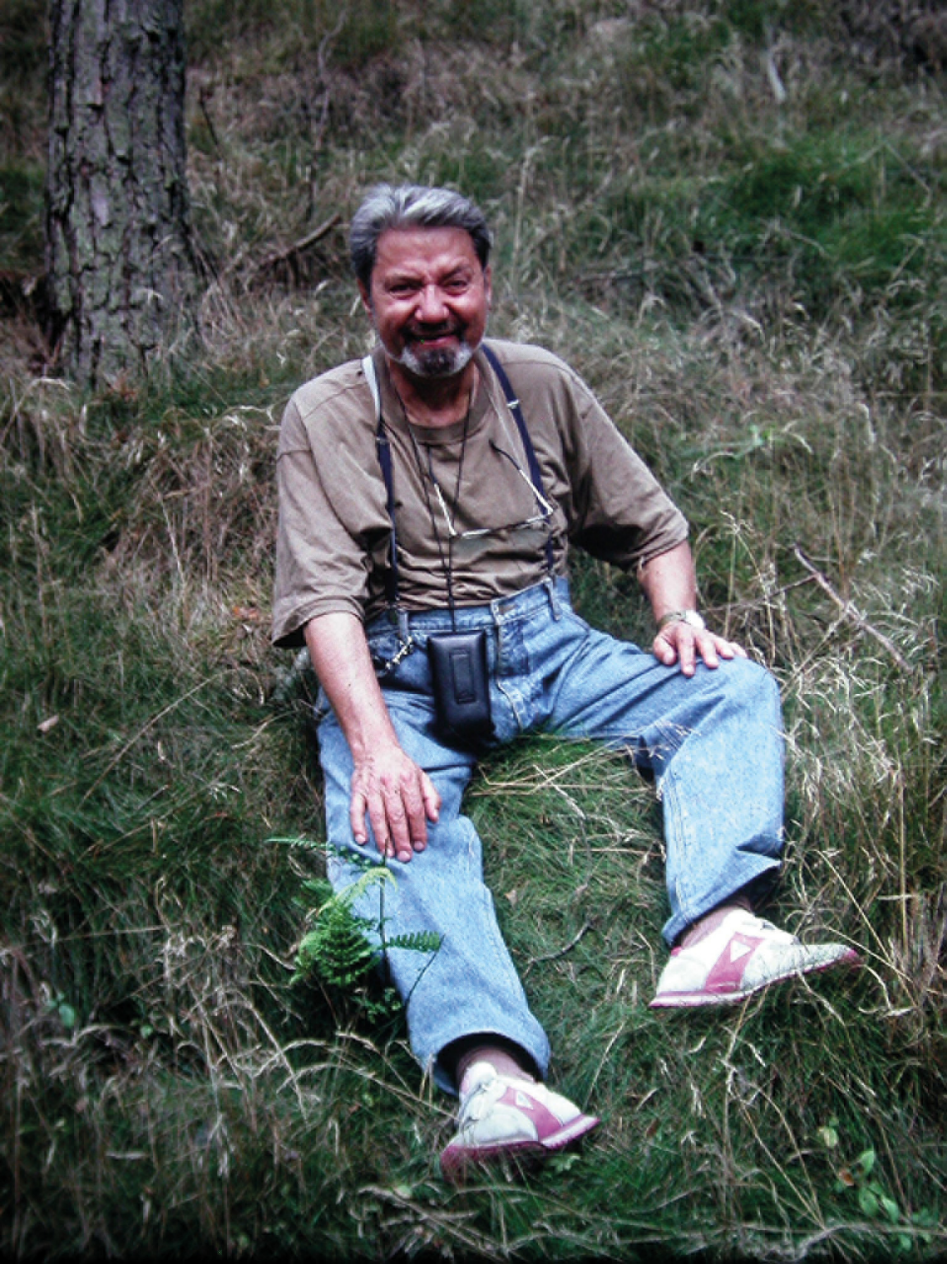
Okmir E. Agakhanjanz. Senne-area near Bielefeld, Germany (Photo: Breckle, 23.7.1992).

The Western Pamirs of Tajikistan constitute an area of high biodiversity with 1,500–2,000 vascular plant species, including 160 endemics, that perform important ecosystem functions and services for the region and the adjacent lowlands [1–3]. However, there is strong evidence that land cover and vegetation in the Pamirs are changing with negative impacts on ecosystem properties [4–7]. Particularly after the dissolution of the Soviet Union pressure on natural resources and human induced land cover change strongly increased [8]. This is primarily associated with a demand for fuel and agricultural products leading to deforestation and overgrazing. For example, the area of Juniper woods strongly decreased during the last decades [2] and the number of cattle increased since 1990 [9]. Overstocking led to a reduced pasture potential, including the expansion of unpalatable and harmful plants [4]. In contrast to pastures, which cover vast areas of the slopes, arable land and associated villages are limited to narrow river terraces and alluvial fans. In this area, riparian Tugai forests play the dominant role in river discharge regulation and embankment stability, and hence in the protection of soils and infrastructure [2]. These forests are strongly degraded because of the energy crisis after the Soviet breakdown that forced the local population to use the Tugai for fuelwood, leading to an increased vulnerability of arable land [10]. This situation was intensified by increased severity of weather conditions, such as torrential rains [5,11], and a rapid decrease in mass balance and extent of local glaciers, resulting in increased summer run-off of the rivers [11–16], trends which are considered to be linked to climate change. In summary, this led to increased erosive forces and decreased erosion control at the same time. A consistent warming within the next decades [17] will further accelerate this development. The temperature increase might also destabilize harvests and therefore intensify food scarcity [6,18], for example due to increased infestations of insects on fruit trees [5]. Furthermore, it might affect high-altitude species [19,20], which encompass many endemics and medicinal plants [7,21]. These plants become threatened by the upward shift of more competitive species from below, which might cause their decline or even extinction because they are ‘trapped’ on the summit and thus lack an escape route [20,22,23]. The discussed findings indicate that the Western Pamirs are a highly dynamic region where anthropogenic and climatic impacts affected vegetation patterns and ecosystem properties in the last decades. Hence, this area provides an ideal field laboratory for detailed studies on the impact of vegetation and ecosystem change on ecosystem functions and services and on the livelihoods of the people. However, such studies require baseline comparison data from the past. Here, we edited, redrew, and evaluated four unpublished historical vegetation maps and the corresponding field notes of the Soviet geobotanist Okmir E. Agakhanjanz (see section 2 and Fig 1) that cover 5,188 km^2^ of the Western Pamirs’ districts Jazgulom, Rushan, Shugnan and Roshtkala and date from 1958 to 1960. A few other maps are available but not yet evaluated. The purpose of this article is to make the historic vegetation data available to the scientific community and thus preserve a hitherto non available and up to now neglected or forgotten data source with great potential for studies on vegetation and ecosystem response to global change [24,25]. For some vegetation units, where it is feasible, we give an estimation of recent developments based on own observations.

## Okmir Agakhanjanz and the History of the Vegetation Maps

“I am a geobotanist. I investigate the plant cover of the Central Asian Mountains and prepare vegetations maps. Attentively I study the plants. I am interested to know how they form communities among themselves and how they thrive in their mountainous environment. At which altitudes do they grow? What kind of slopes and soils do they colonize? And why specifically those?” ([26], p. 7–8).

Prof. Dr. Okmir E. Agakhanjanz (* 5 January 1927 in St. Petersburg; † 28 October 2002 in Minsk; Fig 1) started his geobotanical career on the Taimyr-Peninsula in 1946. Since 1949 he lived in Dushanbe (Tajik SSR) and was member of the Department of Ecology and Experimental Geobotany at the Academy of Sciences of the Tajik SSR. His main duty was to do geobotanical mapping in various parts of the Soviet Union during many self-organized expeditions. The difficulties and the special circumstances of many of these expeditions during Soviet times are described in Agachanjanz [27]. The main goals of the geobotanical mapping expeditions were to establish sound data on grazing potential and biomass production of the natural vegetation in Darwaz, the Fergana Valley, in Southern Tajikistan and predominantly in the Pamirs. From the latter area several geobotanical maps were produced. Vegetation types were characterized and indicated as polygons on the maps. Some of those maps got lost after Prof. Agakhanjanz moved from Dushanbe to Kaliningrad in 1968 and to Minsk in 1972. Some other original maps, including the four we present in this article, are still available and were dedicated to and deposited at the Department of Ecology, headed by Prof. S.-W. Breckle, at the University of Bielefeld, where Prof. Agakhanjanz spent six months as DFG-guest-professor in 1992.

## Study Area

**Fig 2:**
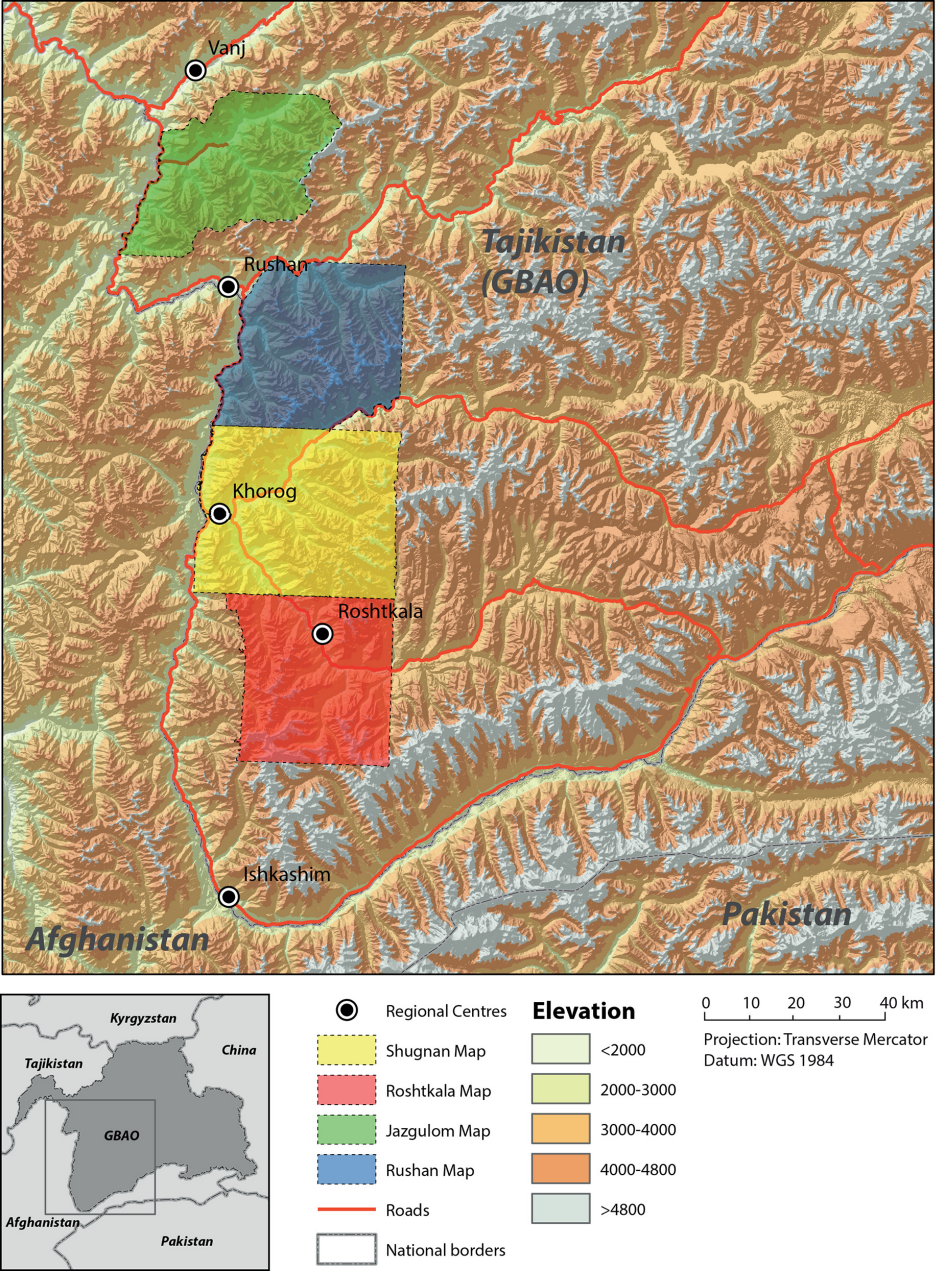
Overview map of the Western Pamirs and the coverage of the four historical vegetation maps.

The Western Pamirs are located in the east of Tajikistan, in the Gorno Badakhshan Autonomous Oblast (GBAO). The four maps discussed in this article cover 5,188 km^2^ of the districts Jazgulom, Rushan, Shugnan and Roshtkala, approximately between 37°N/71°21’E and 38°22’N/72°E (see Fig 2). Elevations range from less than 1,600 m asl in deeply incised valleys up to 6,231 m asl (Peak Vudor). The climate is strongly continental and mainly characterized by the influence of the Westerlies bringing precipitation in winter whereas the summer is dry. Monsoonal influences are assumed to be blocked by the mountain ranges of Hindu Kush and Karakoram. Nevertheless, in summer minor rainfall occurs, which might be related to monsoonal dynamics [28,29]. Walter and Breckle [30] determined the annual mean precipitation within a range of 90 to 217 mm per year. However, the amount of precipitation shows great local differences that are mainly linked to elevation and aspect. It can reach more than 500 mm per year near the snow line at 4,000 m asl, or it can be below 100 mm per year in shielded valleys [11,30,31]. The annual mean temperature ranges between 0.2 and 1.6° C [4,32,33].

## Material and Methods

The original hand-drawn maps were scanned, georeferenced, and digitized. Then, the corresponding land cover class was assigned to each polygon. In order to test for spatial accuracy and to eliminate allocation errors we carried out GPS-based field spot checks for 60 polygons.

Then, the polygons were used to extract pixel based values of the variables elevation, slope, north-exposedness, and east-exposedness with a spatial resolution of 90 m, derived from the Shuttle Radar Topography Mission (SRTM) digital elevation model (DEM) [34]. Aspect, as a circular variable, was transformed to north- and east-exposedness (i.e. cosine and sinus of aspect, see [35]). For discussion and comparison of the values (particularly with elevation values given in the original map legends and in Agachanjanc [31]) minimum, maximum, arithmetric mean, and median were calculated.

Furthermore, we applied generalized additive models (GAMs, [36]) to calculate response curves of the land cover classes and to explore vegetation-topography relationships quantitatively. GAMs are an extension of generalized linear models (GLMs, [37]) that allow for more complex response shapes than a linear one and hence for ecologically more meaningful environmental gradients. We evaluated the results of the GAMs based on the D^2^ value (100 * (null deviance—deviance)/null deviance), which represents the percentage of deviance explained and is analogous to the R^2^ as produced by simple linear regression [38]. The GAMs were fitted using the function *gam* from the *mgcv* R-package [39] with logit as the link function, binomial error distribution, and smoothed spline fits with two degress of freedom. Plant species were named according to the original map legends. Subsequently, the names were checked for validity with the Vascular plants of Russia and adjacent states [40], the Afghan checklist of vascular plants [41], and by expert W. B. Dickoré. Outdated names were supplemented by the new accepted name mentioned in square brackets behind the original name. Still, few names are under dispute, we then use both names, not indicating which the synonymy is.

## Description and Discussion of the Mapped Land Cover Classes

**Fig 3:**
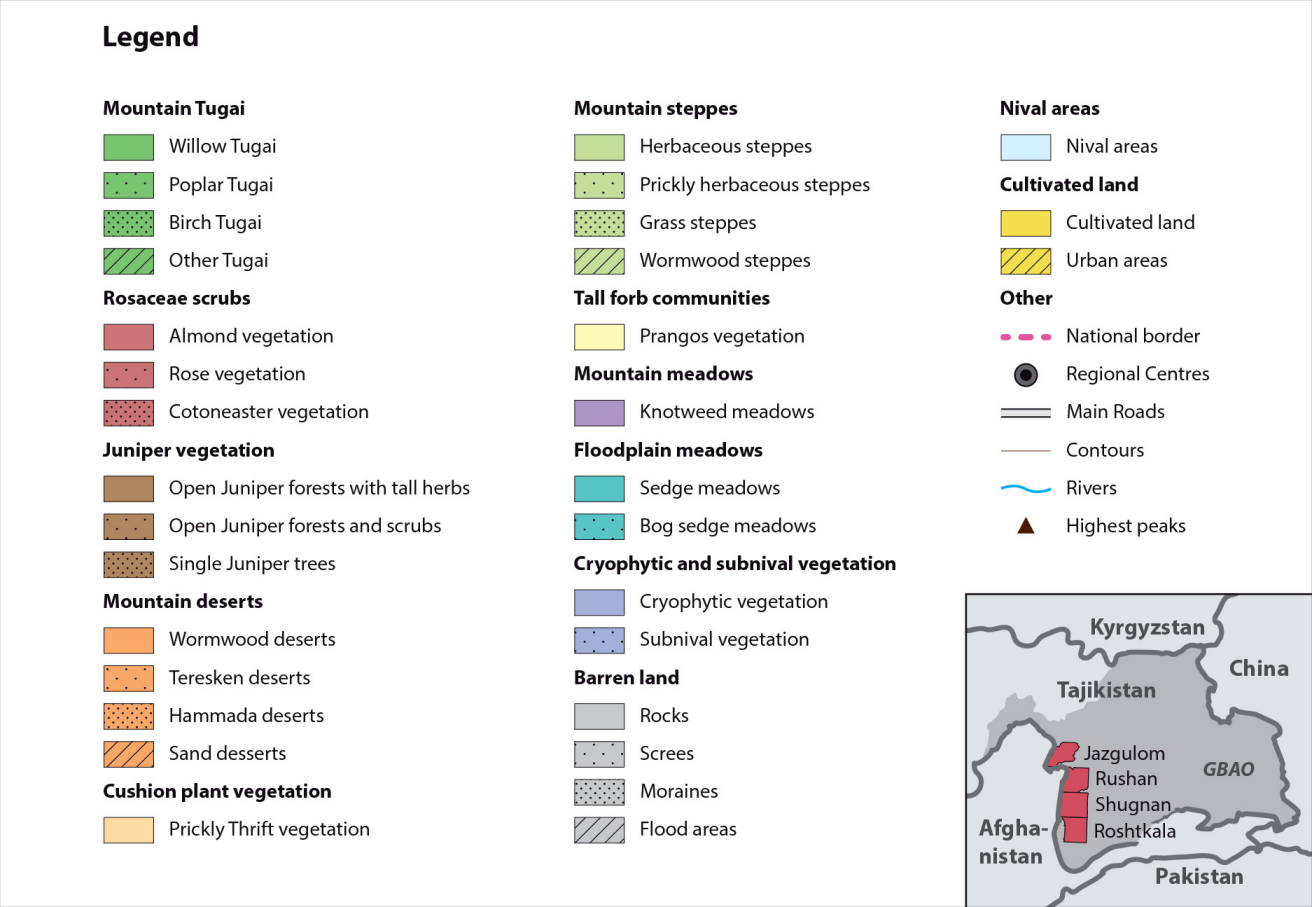
Legend of the land cover classes used in the maps.

**Fig 4:**
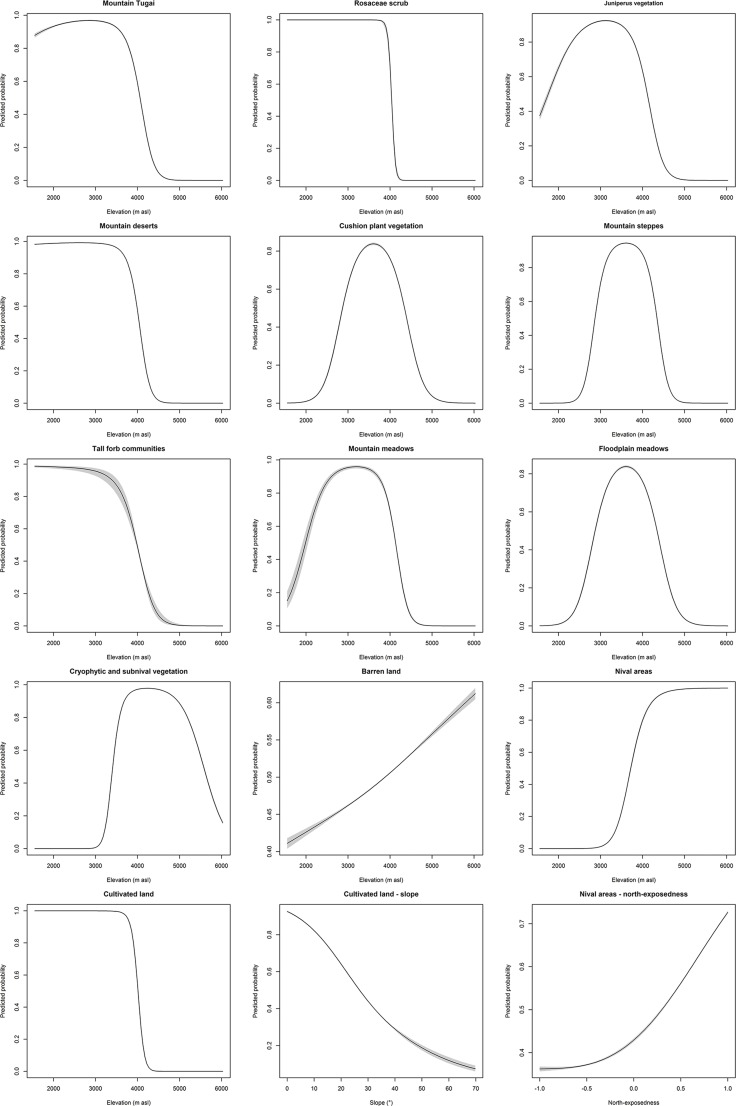
Shape of the response curves. Determined by smoothed spline fits between the predicted probability of occurrence of land cover class presence (y-axis) and elevation (x-axis; the last two graphs refer to slope and north-exposedness) estimated by a GAM using binomial distribution (logit link) for binary data. Shaded areas indicate the 95% confidence bands.

In this section, we present and discuss the digitized vegetation maps, the associated descriptions, and information on the altitudinal distribution, slope and aspect. Agakhanjanz mapped altogether 13 major land cover classes (Fig 3) for an area of 5,188 km^2^ that consists of 2,216 polygons (i.e. spatially coherent patches or biotopes). These classes were further divided in various subunits. In total, the descriptions of these subunits list 252 different plant species. The polygons were used to extract the information on the four topographic variables under consideration summing up to 640,489 grid cells (i.e. extracted values). Some artefacts in the DEM were masked so that finally 634,999 grid values (from 5,143.5 km^2^) were used in the analysis. The shapes of the resulting spline fits for the topographic covariates are shown in Fig 4. A detailed compilation of the results is given in Table 1.

**Table 1:** Description of the land cover classes according to area, elevation, slope, north-exposedness and east-exposedness.

**land cover class**	**area**		**elevation**					**slope**				
	km^2^	%	min	mean	med	max	D^2^	min	mean	med	max	D^2^
**Mountain Tugai**	67.6	1.3	1787	2977	3006	4505	15.1	0.1	22.3	22.8	65	2.7
**Rosaceae scrub**	3.3	<0.1	2087	2716	2698	3535	18.4	3.1	30.4	29.9	48.3	1.4
**Juniper vegetation**	132.5	2.6	1929	3254	3265	4607	13.4	0.8	34.7	35.5	57.3	5.5
**Mountain deserts**	350	6.8	1553	2894	2917	4367	28.8	0.1	28.6	29.2	63.5	0.4
**Cushion plant veg.**	342	6.6	2303	3580	3582	4605	8.2	0.7	27.6	28	64.4	5.8
**Mountain steppes**	206	4	1992	3664	3701	4691	16.2	0.3	26.7	27.2	55.2	2.6
**Tall forb comm.**	3	<0.1	2178	2754	2631	3930	12.4	0.8	27.1	26	47.9	1.5
**Mountain meadows**	12.8	0.3	2079	3276	3335	4220	10.5	0.2	28.9	29.1	55.8	1.1
**Floodplain meadows**	94.0	1.8	1729	3695	3667	4616	8.2	0.1	19.5	19.8	54.7	5.8
**Cryo./subn. vegetation**	696.9	13.4	2748	4272	4278	5254	17.8	0	23.7	24.3	61.9	4.4
**Barren land**	2705	52.1	1554	3874	4018	5544	0.3	0	30.3	31.7	69.8	3.9
**Nival areas**	394.3	7.6	3069	4679	4673	6029	31.7	0.2	24.3	23	67.2	2.5
**Cultivated land**	133.5	2.6	1596	2560	2570	3930	34.6	0	18.2	17.6	58.2	9
**land cover class**	**area**		**north-exposedness**					**east-exposedness**				
	km^2^	%	min	mean	med	max	D^2^	min	mean	med	max	D^2^
**Mountain Tugai**	67.6	1.3	-1	0.3	0.5	1	1	-1	0.1	0.3	1	1
**Rosaceae scrub**	3.3	<0.1	-1	0.6	0.7	1	4	-1	-0.5	-0.6	0.9	3.7
**Juniper vegetation**	132.5	2.6	-1	0	0	1	0.2	-1	-0.1	-0.2	1	0.2
**Mountain deserts**	350	6.8	-1	0.1	0.2	1	0.1	-1	0	0	1	<0.1
**Cushion plant veg.**	342	6.6	-1	0.1	0.1	1	0.3	-1	0	0.1	1	1.8
**Mountain steppes**	206	4	-1	0.1	0.2	1	0.1	-1	0.1	0.3	1	0.8
**Tall forb comm.**	3	<0.1	-1	-0.1	0	1	3.3	-1	0.7	1	1	8.6
**Mountain meadows**	12.8	0.3	-1	0.1	0.2	1	0.2	-1	0.1	0.3	1	0.3
**Floodplain meadows**	94.0	1.8	-1	0	0.1	1	0.3	-1	0.2	0.3	1	1.8
**Cryo./subn. vegetation**	696.9	13.4	-1	0	-0.1	1	0.2	-1	0.1	0.1	1	0.4
**Barren land**	2705	52.1	-1	0	-0.1	1	0.7	-1	-0.1	-0.3	1	1.1
**Nival areas**	394.3	7.6	-1	0.4	0.7	1	4.5	-1	0	0	1	1.1
**Cultivated land**	133.5	2.6	-1	0.1	0.2	1	0.1	-1	0.1	0.2	1	0.2

The D^2^ value indicates the percentage of deviance explained by the environmental variables.

We found a relatively strong and significant relationship between elevation and 12 out of 13 land cover classes (D^2^ between 0.3 and 34.6%, p<0.0001, see Table 1). Slope was important for the distribution of cultivated land (D^2^ = 9.0%). Furthermore, Juniper vegetation, cushion plant vegetation, and floodplain meadows showed D^2^ values of nearly 6%. Aspect, regardless of wether north- or east-exposedness, showed only minor relation to the distribution of the land cover classes. Only nival areas respond to north-exposedness (D^2^ = 4.5%). Furthermore, tall forb communities and Rosaceae scrub show relatively high values, however this result is influenced by spatial autocorrelation due to the low number of polygons representing these land cover classes.

### Mountain Tugai

**Fig 5:**
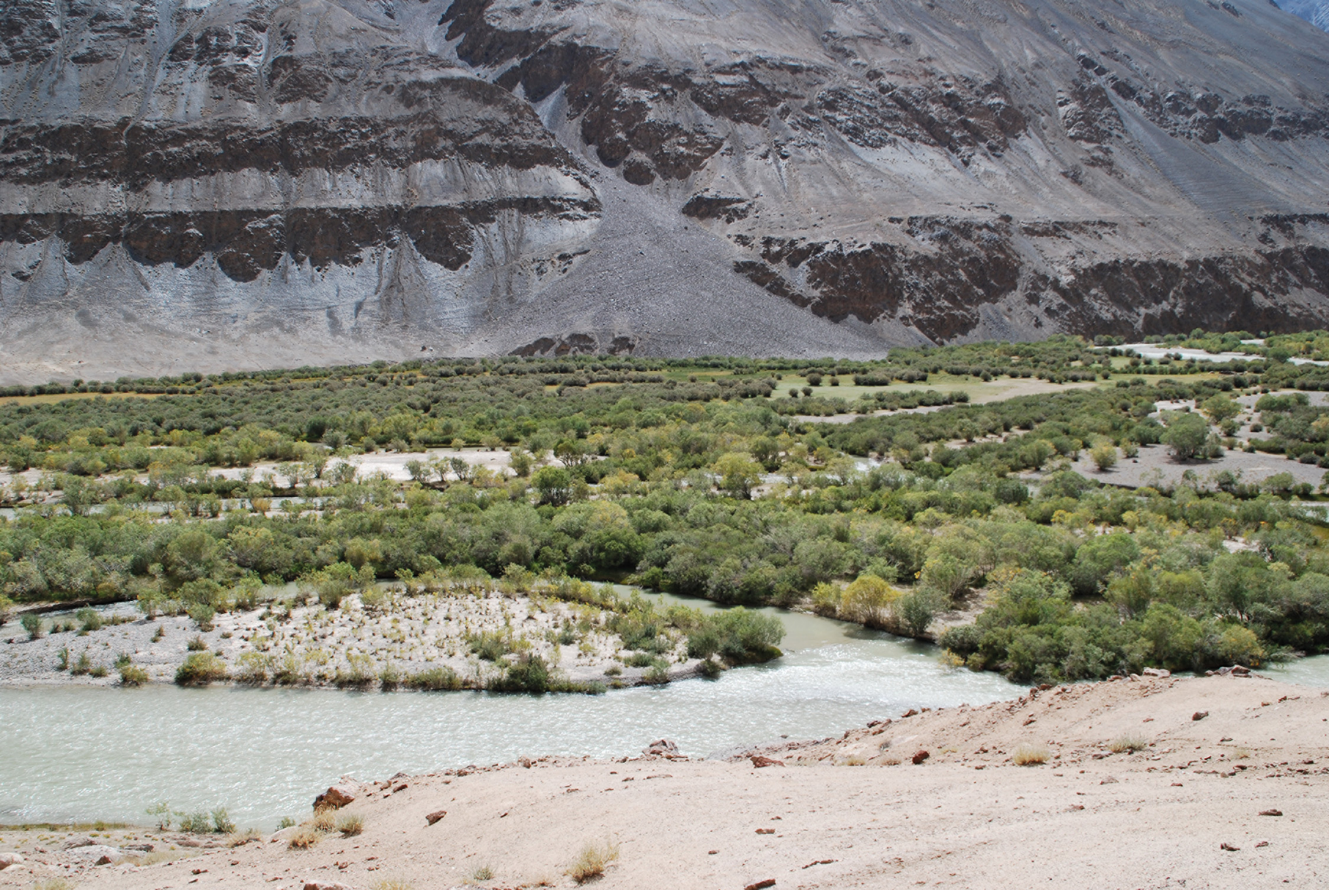
Mountain Tugai predominantly composed of *Salix* spp. and *Hippophaë rhamnoides*. Madian valley, Murghab district, ca. 3,500 m asl. (Photo: Vanselow, 29.8.2011).

Tugai is the local name of Central Asian alluvial scrub and forest (Fig 5). Various woody species can dominate and therefore form different ‘complexes’. Most important are species of willow (*Salix*), birch (*Betula*), poplar (*Populus*), and sea buckthorn (*Hippophaë)*. Tugai vegetation occurs, rather locally, in all investigated areas and covers 67.6 km^2^ (1.3%) of the mapped area. According to the map legends, this formation occurs up to 3,700 m asl. The statistical analysis shows elevations between 1,787 and 4,505 m asl, with an average of 3,036 m asl (median 3,059). The response curve indicates a high probability of occurence from the lowest elevations in the study area up to 3,500 m asl and then sharply drops. Tugai forests degraded in the aspect of area and structure especially after the Soviet breakdown, when they were used for firewood. Meanwhile, however, the situation improved due to programs of communal forest management [10].

#### Willow Tugai

Most widespread Tugai type. Dominated by scrubs of different willow species (e.g. *Salix schugnanica*, *S*. *turanica*, *S*. *rosmarinifolia*, *S*. *wilhelmsiana*, *S*. *coerulea [S*. *capusii]*). Associated with *Hippophaë rhamnoides*, *Populus pamirica*, *Lonicera stenantha*, *Rosa fedtschenkoana*, and rich grass and herb meadows (with e.g. *Roegneria schugnanica [Elymus schugnanicus]*, *Hordeum bogdanii*, *Oxytropis gorbunovii*, *Nevskiella gracillima [Bromus gracillimus]*, *Glycyrrhiza glabra*, *Orchis umbrosa [Dactylorhiza umbrosa]*, *Agrostis alba [A*. *gigantea]*. On the banks of Panj River also associated with *Myricaria alopecuroides [M*. *bracteata]*.

**Fig 6:**
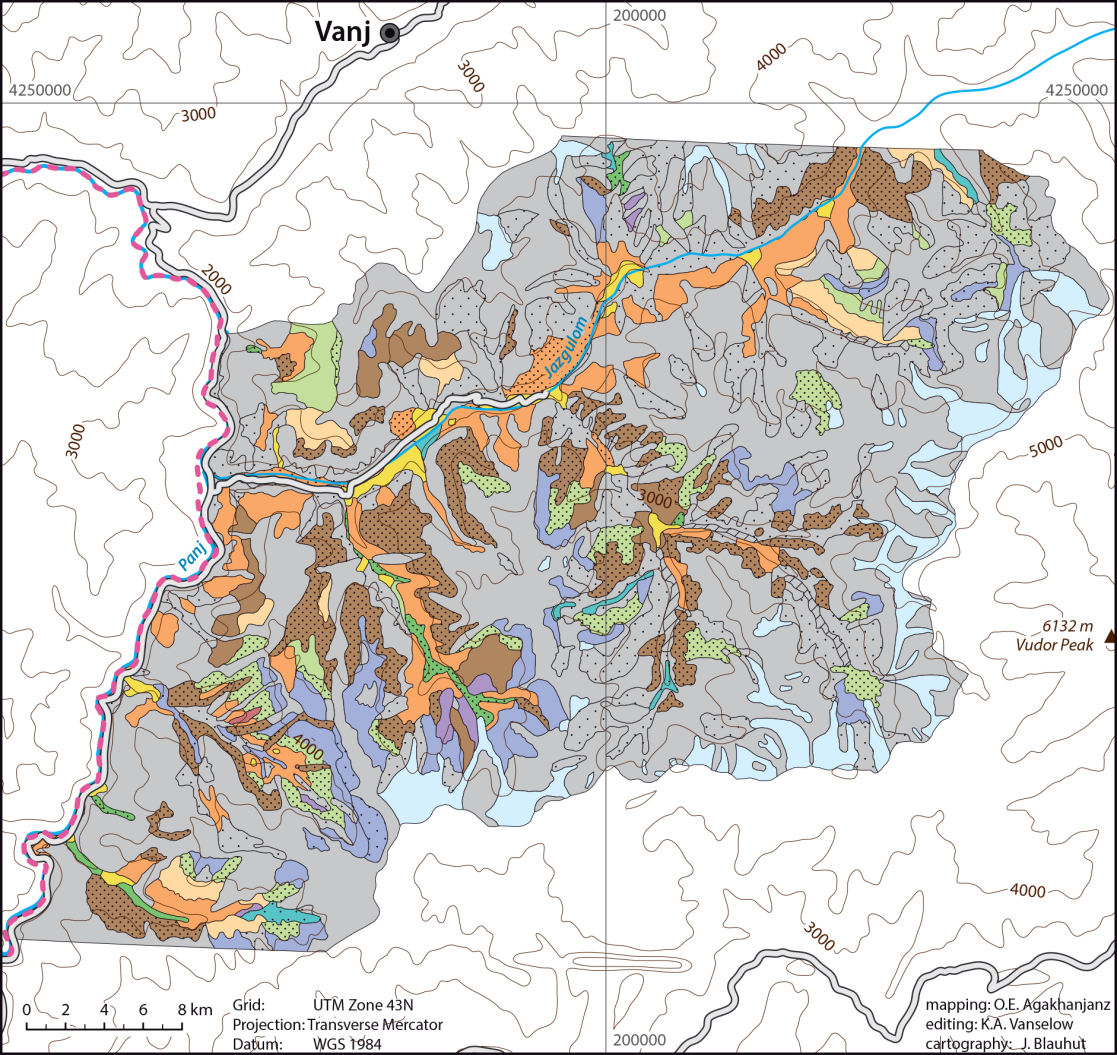
Vegetation map of the Jazgulom area.

In the Jazgulom area (Fig 6) willow species form floodplain and gallery forests. *Salix turanica* frequently grows up to 15 m tall and covers 70 to 80%. Other *Salix* species form a second lower layer. A third layer consists of scrubs (*Ribes janczewskii*, *Lonicera* species), tall grasses (*Agrostis alba [A*. *gigantea]*, *Calamagrostis dubia [C*. *pseudophragmites]*), and herbs (*Aquilegia lactiflora*, *Incarvillea olgae*, *Equisetum arvense*, *Silene commutata [Oberna commutata]*, *Salvia sclarea*).

#### Birch Tugai

Dominated by *Betula pamirica* (in some places also *B*. *procurva*) that grow up to 18 m tall. Characterized by several vegetation layers. Often associated with *Hippophaë rhamnoides*, *Salix rosmarinifolia*, *S*. *schugnanica*, *S*. *turanica*, or if lower with *Rosa webbiana*, *Lonicera microphylla*, *L*. *korolkowii*, *Berberis integerrima*, and *Cotoneaster multiflorus*. On gravelly soils associated with *M*. *alopecuroides [Myricaria germanica*, *M*. *bracteata]* and *Ribes janczewskii*.

#### Poplar Tugai

Dominated by *Populus pamirica*. Often associated with *Hippophaë rhamnoides*, *Salix turanica*, *S*. *schugnanica*, *S*. *rosmarinifolia*, and scrubs (*Rosa fedtschenkoana*, *Lonicera stenantha*, *Ribes janzcewskii*, *R*. *meyeri*). Sometimes associated with *Betula pamirica* and herbs and grasses (e.g. *Polygonum coriarium [Aconogonon coriarium]*, *Glycyrrhiza glabra*, *Malacurus lanatus [Leymus lanatus]*, *Poa bucharica*, *Carex griffithii [C*. *nivalis]*, *Orchis umbrosa [Dactylorhiza umbrosa]*).

#### Other Tugai

**Fig 7:**
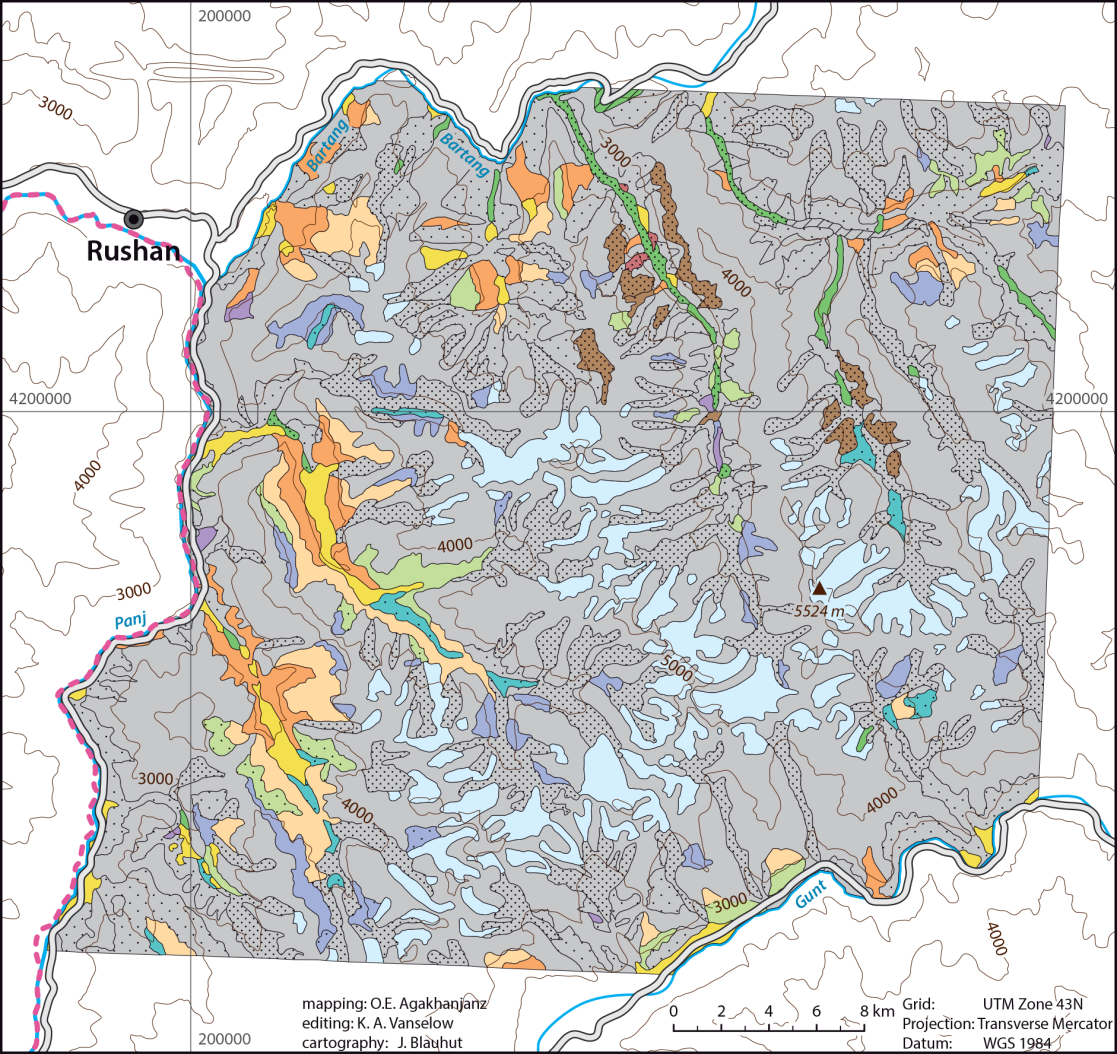
Vegetation map of the Rushan area.

Tugai dominated by sea buckthorn (*Hippophaë rhamnoides*) associated with *Tamarix ramosissima* and open grass vegetation. On alluvial sands and screes in the Rushan area (Fig 7).

**Fig 8:**
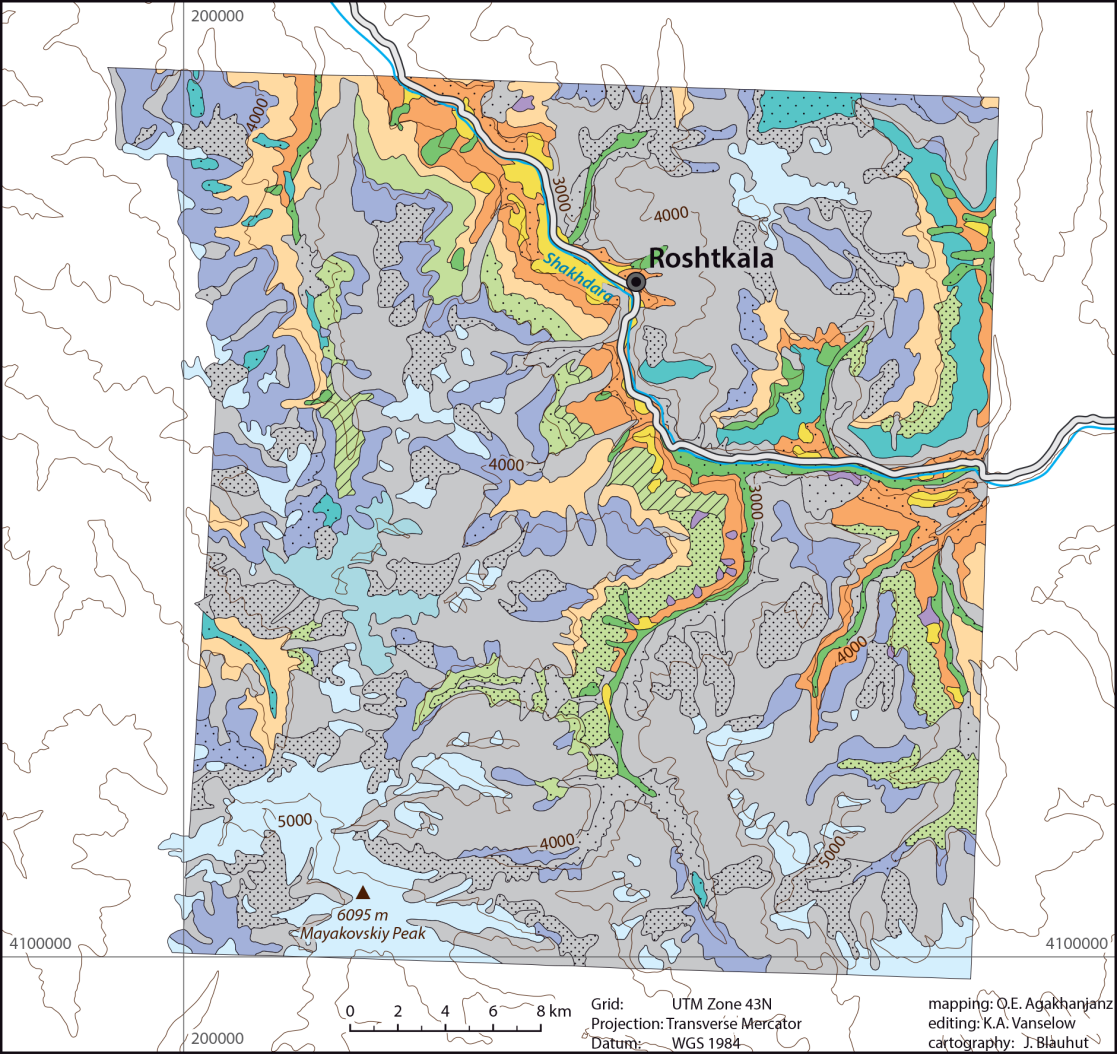
Vegetation map of the Shugnan area.

Currant vegetation with *Ribes janzcewskii* associated with *Polygonum coriarium [Aconogonon coriarium]* and *Rosa fedtschenkoana*. Near springs and river inlets in the Roshtkala area (Fig 8).

### Rosaceae scrub

Rosaceae scrubs cover only very small areas of the study area (3.3 km^2^; <0.1%) and consist of almond, rose, and cotoneaster vegetation. According to the map legends and Agachanjanc [31] they occur from 2,500 up to 2,900 m asl, topographic analysis shows occurrences between 2,087 and 3,535 m asl with a mean at 2,716 m asl (median 2698). The response curve shows a very high probability of occurrence up to 4,000 m asl and, thereafter, a steep decline towards zero probability.

#### Almond vegetation

Scrubs, dominated by shrubs or small trees of *Amygdalus bucharica* associated with tall herbs (*Eremurus stenophyllus [E*. *ambigens]*, *Incarvillea olgae*, *Silene commutata [Oberna commutata]*, *Lindelofia macrostyla [L*. *anchusoides]*, *Helichrysum mussae*, *Leptorhabdos micrantha [L*. *parviflora]*, *Ferula jaeschkeana [F*. *kuhistanica]*) and other shrubs (*Rosa webbiana*, *Lonicera persica [L*. *nummularifolia]*, *Ziziphora bungeana [Z*. *clinopodioides]*). Limited to narrow gullies in the north of the Western Pamirs.

#### Rose vegetation

Composed of *Rosa kokanica* or *Rosa webbiana* associated with *Exochorda alberti [E*. *korolkowii]*, *Lonicera korolkowii* and tall herbs (e.g. *Polygonum coriarium [Aconogonon coriarium]*, *Prangos pabularia*).

#### Cotoneaster vegetation

Composed of *Cotoneaster uniflorus* and *C*. *multiflorus* associated with *Lonicera microphylla*. Occurs on alluvial fans with abundant amounts of snow.

#### Juniper vegetation

Juniper vegetation occurs in 132.5 km^2^ (2.6%) of the study area in elevations between 1,929 and 4,607 m asl (mean 3,254, median 3,265). The probability of occurrence is very high between 2,500 and 4,000 m asl and sharply drops for elevations below and above that values. According to the map legends Juniper vegetation is abundant up to 3,800 (rarely 4,000) m asl, Agachanjanc [31] states a distribution range between 2,900 and 3,500 m asl. There is a maximum probability of occurrence between 40 and 50° slope angle. In contrast, flat and very steep slopes show a low probability. Three different classes of juniper vegetation can be defined: Open Juniper forests with tall herbs, Open Juniper forests and scrubs, and Single Juniper trees. However, according to the authors’ observations, there are nearly no open forests left, at least in Rushan, Shugnan, and Roshtkala district. These formations turned into grass or herb dominated steppes with some very scattered trees and shrubs.

#### Open Juniper forests with tall herbs

Open Juniper forests, with *Juniperus seravschanica [J*. *polycarpos]* associated with *Prangos pabularia*, *Ferula jaeschkeana [F*. *kuhistanica]*, *Hedysarum flavescens*, *Polygonum coriarium [Aconogonon coriarium]*, *Rosa kokanica*, and sometimes *Astragalus* species.

#### Open Juniper forests and scrubs

Dominated by *Juniperus seravschanica [J*. *polycarpos]*, rarely *Juniperus sibirica [J*. *communis* subsp. *nana]* or *Juniperus schugnanica [J*. *semiglobosa]* (the latter only in the Roshtkala area). A characteristic feature of this vegetation type is a distinct shrub layer with *Rosa kokanica*, *Rosa maracandica*, *Rosa korshinskiana*, *Lonicera korolkowii*, and near springs *Betula pamirica*. Furthermore, associated with dwarf shrubs and herbs (e.g. *Artemisia persica*, *Artemisia lehmanniana*, *Cousinia pannosa*, *Cousinia rubiginosa*, and on screes *Acantholimon korolkovii*, *Acantholimon parviflorum*) and grasses (*Stipa caucasica*, *Stipa bella [S*. *drobovii]*, *Stipa kirghisorum*, *Poa relaxa*). Small patches of this association are widespread in higher altitudes (up to 3800 m asl). On conglomerate slopes and on rocks in the Roshtkala area (Fig 8) also associated with steppe elements (*Stipa caucasica*, *Stipa szowitsiana [S*. *caspia]*, *Artemisia korshinskyi*, *Helictotrichon fedtschenkoi*).

#### Single Juniper trees

Formed by single trees of *Juniperus seravschanica [J*. *polycarpos]* on rocks up to 4000 m asl. Those might be regarded as very old relict trees.

### Mountain deserts

Mountain deserts cover 350 km^2^ (6.8%) of the mapped area and show occurrences between 1,553 and 4,367 m asl (mean 2,894, median 2,917). The response curve indicates a very high probability of occurrence up to 3,500 m asl and, thereafter, a sharp drop of the curve that reaches zero probability at 4,500 m asl. Agachanjanc [31] states a range from 2,000 to 3,500 m asl, the map legends give values between 2,500 and 3,400 m asl. Mountain deserts can be divided into four main types.

#### Hammada deserts

Open *Hammada wakhanica [Haloxylon griffithii]* deserts grow on dry plateaus and terraces. Vegetative ground cover 10 to 15%. Sometimes associated with *Artemisia vachanica*, *Artemisia porrecta*, *Salsola pestifer [S*. *australis]*, *Salsola montana*, *Girgensohnia oppositiflora*, *Anisantha tectorum [Bromus tectorum]*, *Atriplex moneta*, *Lactuca orientalis [Scariola orientalis]*, *Silene samarkandensis*, and in lower elevations with ephemeral plants (*Carex pachystylis*, *Poa bulbosa*, rarely also *Peganum harmala*, *Macrotomia euchroma [Arnebia euchroma]*, *Echinops nanus*).

#### Teresken deserts

**Fig 9:**
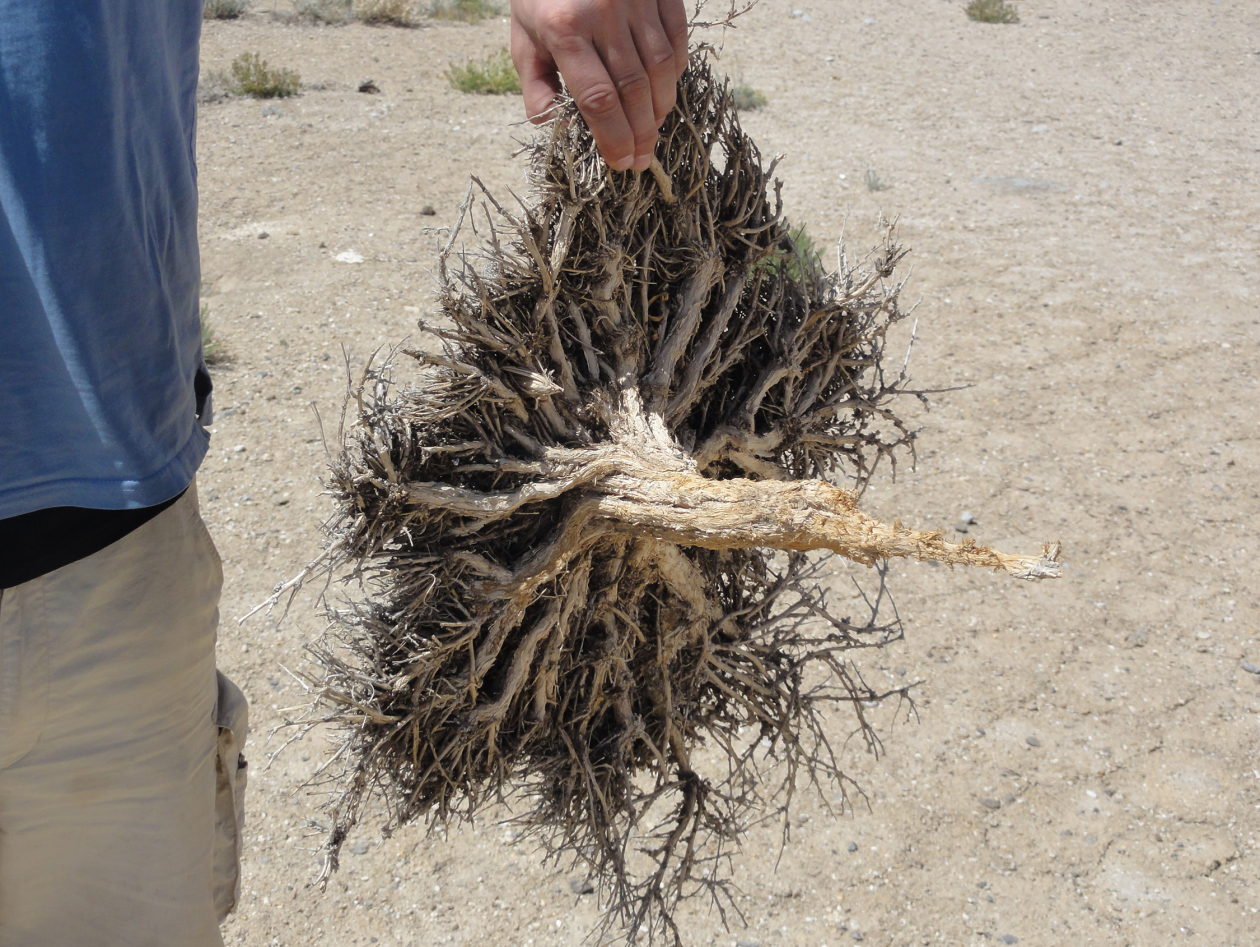
Teresken (*Krascheninnikovia ceratoides*). Madian valley, Murghab district, 4,110 m asl. (Photo: Samimi, 24.8.2011).

**Fig 10:**
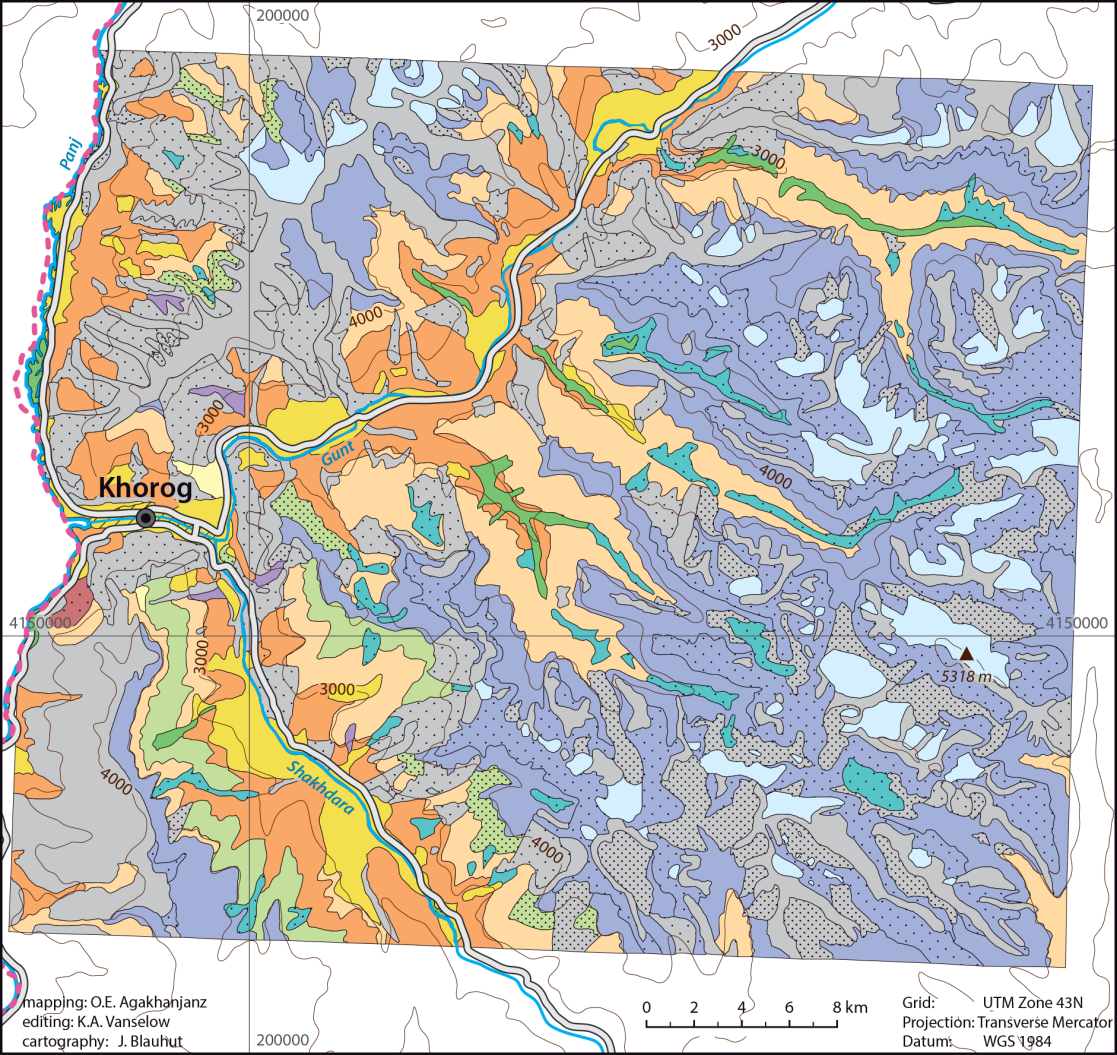
Vegetation map of the Shugnan area.

Characterized by Pamirian Winterfat dwarf shrubs (*Ceratoides papposa [Krascheninnikovia ceratoides]*, see Fig 9 and [30], p. 424 for more information on this species). Local name Teresken. In the Roshtkala area (Fig 8) distributed in scattered associations. At lower elevations (around 2,500 m asl) with *Artemisia vachanica*, *Lagochilus seravschanicus*, *Ephedra tibetica [Ephedra intermedia]*, *Ephedra gerardiana*, *Stipa badachschanica*, *Echinops maracandicus*, *Kochia prostrata*, *Acantholimon parviflorum*; in the higher parts (2,600 to 3,200 m asl) on fine-grained soils associated with *Stipa orientalis*, *Astragalus tragacantha*, *Ziziphora bungeana [Ziziphora clinopodioides]*, *Nepeta podostachys*, *Nepeta badamdarica [N*. *glutinosa]*, *Centaurea squarrosa*, *Hyoscyamus pusillus*, *Cousinia rubiginosa*, *Bromus* spp. In the Shugnan area (Fig 10) associated with *Artemisia vachanica*, *Ephedra tibetica [Ephedra intermedia]*, *Acantholimon parviflorum*. On scree slopes also with *Acantholimon korolkovii*. Sometimes also associated with *Artemisia korshinskyi* and *Salsola montana*.

#### Wormwood deserts

Four different types can be differentiated: Typical *Artemisia vachanica* desert associated with *Artemisia korshinskyi*. Sometimes also associated with *Ceratoides papposa [Krascheninnikovia ceratoides]*, *Ephedra tibetica [E*. *intermedia]*, *Lactuca orientalis [Scariola orientalis]*, *Kochia prostrata*, *Lindelofia macrostyla [L*. *anchusoides]*. On fine-grained soils dominated by ephemeral plants (*Poa bulbosa*, *Carex pachystylis*). On screes with *Acantholimon korolkovii*. Frequently with steppe elements (*Stipa caucasica*, *Stipa szowitsiana [S*. *caspia]*, *Stipa badachschanica*, *Stipa orientalis*, *Cousinia rubiginosa*, *Artemisia lehmanniana*).

Steppe-like wormwood deserts, equally dominated by *Artemisia vachanica* and *Artemisia korshinskyi* associated with *Artemisia persica*, *Piptatherum purpurascens*, *Stipa badachschanica*, *Stipa caucasica*, *Poa relaxa*, *Poa bactriana*, *Cousinia rubiginosa*, *Allium jacquemontii [A*. *pamiricum]*, *Ferula grigoriewii*, *Lindelofia macrostyla [L*. *anchusoides]*, *Kochia prostrata*, *Eremopoa persica*, *Ziziphora bungeana [Z*. *clinopodioides]*, *Chenopodium botrys [Dysphania botrys]*, *Achillea filipendulina*, *Ephedra tibetica [E*. *intermedia]*, *Andropogon ischaemum [Bothriochloa ischaemum]*. Sometimes associated with *Prangos pabularia*.

Steppe-like wormwood deserts (dominated by *Artemisia vachanica* and *Artemisia korshinskyi*) with mountain xerophytes (e.g. *Acantholimon korolkovii*, *Ephedra gerardiana*, *Astragalus roschanicus*, *Astragalus lasiosemius*, *Arenaria griffithii [Eremogone griffithii]*), prickly herbs (e.g. *Cousinia rubiginosa*), steppe grasses (e.g. *Stipa caucasica*, *Stipa ovchinnikovii*, *Piptatherum vicarium*, *Piptatherum purpurascens*, *Poa relaxa*, *Festuca valesiaca*), and in some places with *Prangos pabularia*.

Typical *Artemisia tenuisecta*-*Artemisia vachanica* steppe desert with ephemeral plants (*Poa bulbosa*, *Carex pachystylis*) associated with *Perovskia scrophulariifolia*, *Scabiosa songarica*, *Origanum tyttanthum [O*. *vulgare* subsp. *gracile]*, *Ziziphora bungeana [Z*. *clinopodioides]*, *Kochia prostrata*. On screes also associated with *Peganum harmala*. Only in the Jazgulom area (Fig 6) up to 2,000 m asl.

#### Sand deserts

Sand deserts with *Myricaria* species, *Trichodesma incanum*, *Lindelofia macrostyla [L*. *anchusoides]*, *Glycyrrhiza glabra*, and *Inula salsoloides [Limbarda salsoloides]* are limited to one small location on an old river terrace along Panj River in the Jazgulom area (Fig 6).

### Cushion plant vegetation

Cushion plant vegetation occurs in 342 km^2^ (6.6%) of the mapped area in elevations between 2,303 and 4,605 m asl (mean 3,580, median 3,582). In Agachanjanc [31] and the map legends 3,000 m asl are stated as the minimum and 4,000 m asl (partly 4,700) as the maximum elevation. The response curve displays a probability peak of 0.8 at 3,800 m asl.

#### Prickly Thrift vegetation

The dominant genus that forms most types of the cushion plant vegetation is *Acantholimon* (Prickly Thrift).

**Fig 11:**
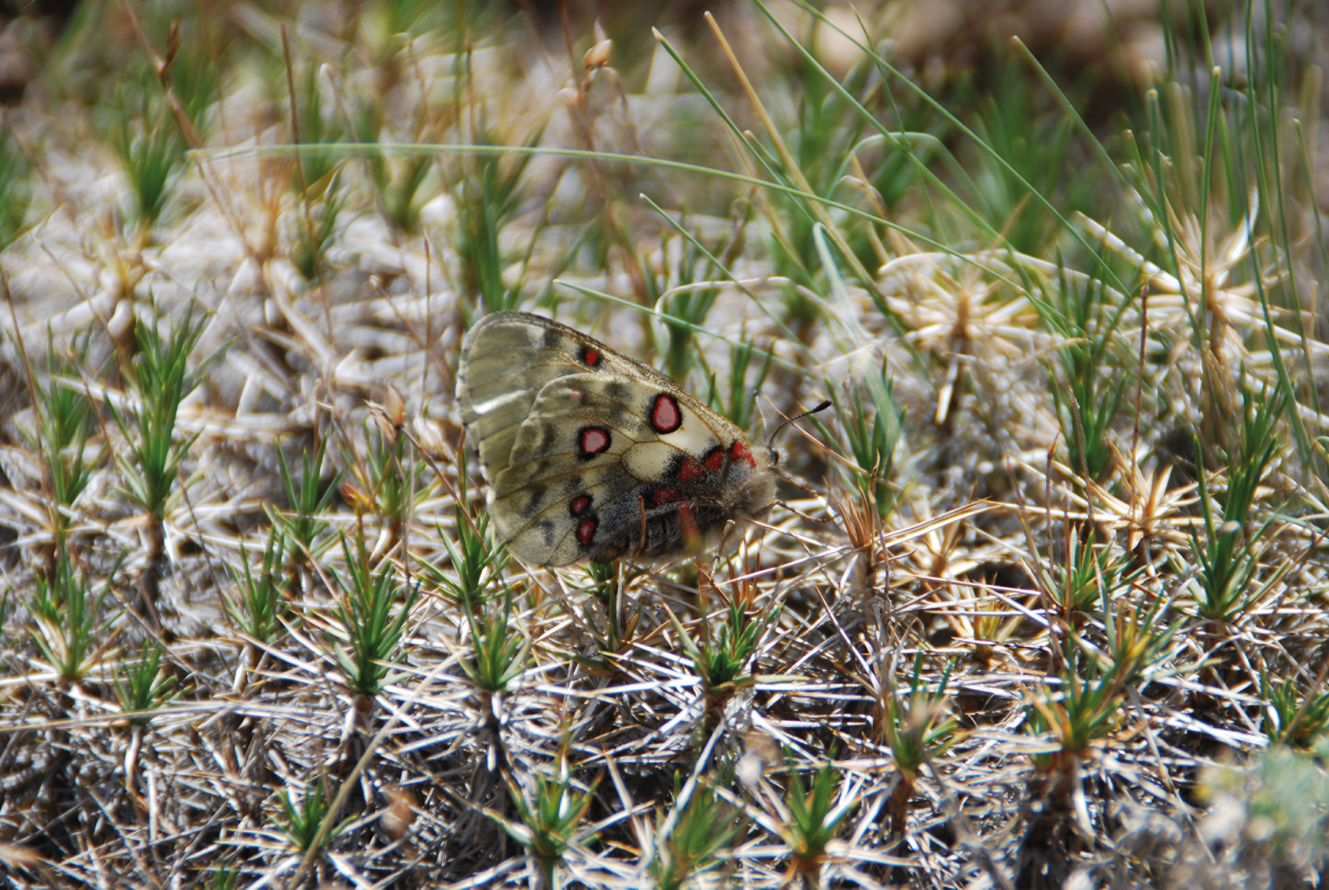
*Acantholimon korolkovii* (with *Parnassius jacquemontii*), a typical species of cushion plant vegetation in the Western Pamirs. Geisev Valley, Rushan district, 3,590 m asl. (Photo: Vanselow, 18.6.2014)

Most extensive type dominated by *Acantholimon korolkovii* (Fig 11) associated with other mountain xerophytes (*Acantholimon alatavicum*, *A*. *pamiricum*, *A*. *parviflorum*, *Astragalus lasiosemius*, *A*. *roschanicus*, *Hedysarum cephalotes [H*. *minjanense]*, *Scorzonera acanthoclada*, *Onobrychis echidna*, *Arenaria griffithii [Eremogone griffithii]*, *Cousinia rubiginosa*, *Trigonella griffithii [Melilotoides gontscharovii]*, *Artemisia lehmanniana*, sometimes with *Ephedra gerardiana*, *Artemisia vachanica*).

Steppic variant rich in grasses (*Stipa caucasica*, *S*. *kirghisorum*, *S*. *szowitsiana [S*. *caspia]*, *Festuca valesiaca*, *Poa relaxa*, *Piptatherum platyanthum*), *Nepeta* species, *Artemisia leucotricha*, *Artemisia korshinskyi*, *Artemisia lehmanniana*.

In some flat areas *Acantholimon korolkovii* associated with *Astragalus fedtschenkoanus*, *Acanthophyllum pungens*, *Lagochilus seravschanicus*, *Cousinia rubiginosa*.

Meadow-like type of *Acantholimon korolkovii* vegetation with open layers of *Prangos pabularia*. High elevations with abundant snow cover of the Rushan area (Fig 7).

**Fig 12:**
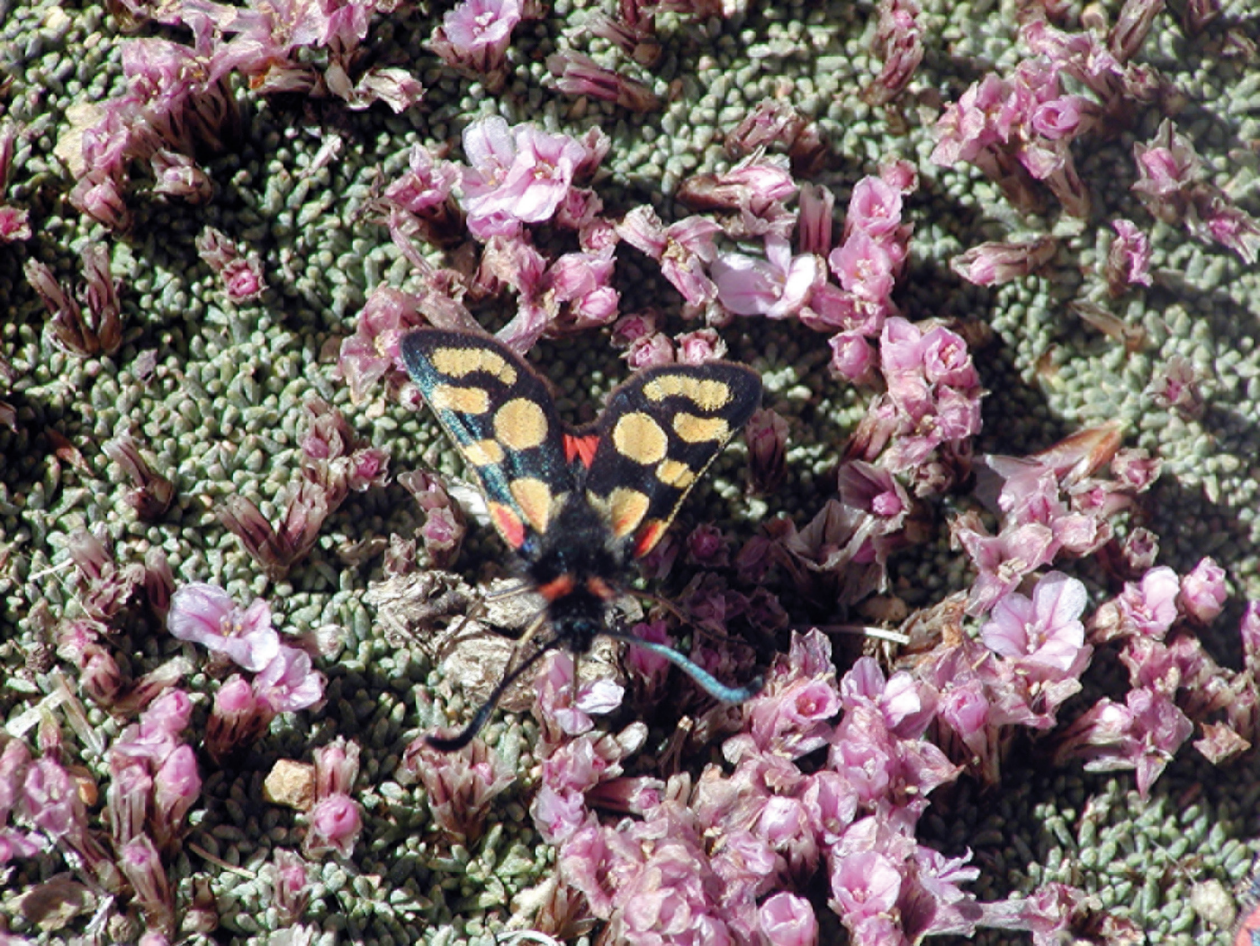
*Acantholimon diapensioides* (with *Zygaena pamira*); South of Turumtai Kul, Shugnan district, 4,230 m asl. (Photo: Naumann, 17.8.2002).

*Acantholimon diapensioides* (Fig 12) deserts associated with *Artemisia korshinskyi*, *Artemisia leucotricha*, *Artemisia rhodantha*, *Oxytropis poncinsii [O*. *stracheana]*, *Hedysarum cephalotes [H*. *minjanense]*, *Xylanthemum pamiricum*. Sometimes associated with *Artemisia lehmanniana*, *Parrya exscapa [Leiospora exscapa]*, *Chorispora macropoda*. Very high elevations (4,350 to 4,700 m asl) in the Shugnan and Roshtkala area (Figs 8 and 10).

*Acantholimon parviflorum* associated with *Ephedra gerardiana*, *Cicer macracanthum*, *Cousinia rubiginosa*, *Poa bactriana*, *Stipa caucasica*, *Artemisia vachanica*. Lower elevations of the Jazgulom area (Fig 6).

*Acantholimon pamiricum* deserts associated with *Artemisia korshinskyi*, *A*. *leucotricha*, *Xylanthemum pamiricum* and sometimes *Stipa orientalis*. Shugnan and Roshtkala area (Figs 8 and 10) up to 4,200 m asl.

*Astragalus roschanicus* associated with *Acantholimon korolkovii*, *Onobrychis echidna*, and sometimes steppe grasses (*Roegneria jacquemontii [Elymus longe-aristatus* subsp. *canaliculatus*, *Elymus jacquemontii]*, *Stipa caucasica*). Only in the Rushan area (Fig 7) up to 4,100 m asl.

### Mountain steppes

Mountain steppes cover 206 km^2^ (4.0%) of the mapped area an occur between 1,992 and 4,691 m asl (mean 3,664, median 3,701), with a probability peak of 0.9 around 3,600 m asl. Agachanjanc [31] states an altitudinal distribution between 3,100 and 4,000 m asl, in the map legends a range from 3,200 to 4,400 m asl is given. Four different steppe types can be differentiated: Grass steppes, Herbaceous steppes, Prickly Herbaceous steppes, and Wormwood steppes. However, according to the authors’ perception, prickly *Cousinia* herbs spread intensively since the completion of Agakhanjanz’s work therefore a differentiation between the two types of herbaceous steppes is not reasonable anymore.

#### Grass steppes

The typical grass steppes of the Western Pamirs are meadow-grass (*Poa*), feather grass (*Stipa*), and fescue (*Festuca*) steppes that are all associated with mountain xerophytes (e.g. *Acantholimon*, *Ephedra*, *Arenaria [Eremogone]*, *Scorzonera* species), desert plants (e.g. *Artemisia* and *Ceratoides [Krascheninnikovia]*) and cryophytic plants.

Feather grass associations with *Stipa badachschanica*, *S*. *caucasica*, and *S*. *orientalis* associated with *Poa relaxa*, *Piptatherum laterale*, *Helictotrichon fedtschenkoi*, *Artemisia lehmanniana*, *Nepeta podostachys*, *Ziziphora bungeana [Z*. *clinopodioides]*. On slopes also associated with *Artemisia korshinskyi*, *A*. *vachanica*, *Cousinia rubiginosa*, *Tragopogon pusillus*, *Ephedra gerardiana*, *Astragalus roschanicus*. Widespread in the Roshtkala area (Fig 8).

Feather grass steppes dominated by *Stipa badachschanica*, *S*. *ovczinnikovii*, *S*. *kirghisorum*, *S*. *rubiginosa*, *S*. *orientalis* associated with *Artemisia tenuisecta*, *A*. *vachanica*, *A*. *persica*, *Piptatherum laterale*, *Poa bactriana*, *Ephedra gerardiana*, *Stipa caucasica*, *Cousinia rubiginosa*, *Zerna turkestanica [Bromopsis turkestanica*, *Bromus paulsenii]*, *Ziziphora bungeana [Z*. *clinopodioides]*. Jazgulom area (Fig 6).

Feather grass steppes dominated by *Stipa caucasica* associated with *Acantholimon* species, *Artemisia lehmanniana*, *Arenaria griffithii [Eremogone griffithii]*, *Scorzornera acanthoclada*, *Cicer songaricum*. Sometimes also associated with *Stipa szowitsiana [S*. *caspia]* and *Poa zapryagajevii*.

*Festuca* steppes with *Festuca alaica* or *F*. *valesiaca* associated with *Stipa* species, *Poa relaxa*, *Piptatherum alpestre*.

Typical meadow-grass steppes with *Poa relaxa* associated with *Zerna turkestanica [Bromopsis turkestanica*, *Bromus paulsenii]*, *Poa marginata [P*. *litvinoviana]*, *Poa bactriana*, *Stipa turkestanica*, *S*. *caucasica*, *Cousinia rubiginosa*, *Ephedra gerardiana*, *Arenaria griffithii [Eremogone griffithii]*. Jazgulom area (Fig 6).

Steppes with *Piptatherum laterale*, *Roegneria ugamica [Elymus dentatus]*, *R*. *jacquemontii [E*. *jacquemontii]*, *Allium gracillimum*, and *Solenanthus olgae*. Jazgulom area (Fig 6) on steep slopes above 3,500 m asl.

*Bromus* steppes associated with *Zerna turkestanica [Bromopsis turkestanica*, *Bromus paulsenii]*, *Poa marginata [P*. *litvinoviana]*, *Rhodiola heterodonta*, *Megacarpaea gracilis*, *Arenaria griffithii [Eremogone griffithii]*, *Ephedra regeliana*, *Piptatherum laterale [Oryzopsis lateralis]*. In higher elevations (above 3,900 m asl) associated with *Roegneria ugamica [Elymus dentatus*, *E*. *nevskii]* and *Poa relaxa*. Jazgulom area (Fig 6).

*Leucopoa olgae [Festuca olgae]* steppes associated with *Stipa* species, *Festuca valesiaca*, *Helictotrichon fedtschenkoi*, *Nepeta pamirensis*, *Cousinia rubiginosa*, *Scorzonera acanthoclada*, *Acantholimon alatavicum*. Sometimes associated with *Onobrychys echidna*. Shugnan and Roshtkala area (Figs 8 and 10) up to 4,200 m asl.

Tall grass steppes composed of *Roegneria ugamica [Elymus dentatus*, *E*. *nevskii]*, *R*. *tianschanica [E*. *tianschanigenus]*, *R*. *interrupta [E*. *praeruptus]*, *R*. *jacquemontii [Elymus longe-aristatus* subsp. *canaliculatus*, *E*. *jacquemontii]*, *Piptatherum vicarium*, *P*. *platyanthum*, *P*. *laterale*, *Zerna turkestanica [Bromopsis turkestanica*, *Bromus paulsenii]*, *Helictotrichon fedtschenkoi*. Associated with *Stipa* species, *Festuca valesiaca*, *Artemisia lehmanniana*, *Nepeta podostachys*, *Ziziphora bungeana [Z*. *clinopodioides]*, sometimes *Cousinia pannosa*. Jazgulom and Rushan area (Figs 6–7) up to 3,900 (sometimes 4,200) m asl.

#### Herbaceous steppes

The most widespread type of herbaceous steppes is composed of *Cousinia rubiginosa* associated with *Stipa* species, *Prangos pabularia*, *Polygonum coriarium [Aconogonon coriarium]*. On screes associated with *Acantholimon korolkovii*, *Onobrychys echidna*, *Astragalus roschanicus*, *Arenaria griffithii [Eremogone griffithii]*, and sometimes open layers of *Juniperus seravschanica [J*. *polycarpos]*. In lower elevations associated with *Acantholimon alatavicum*, *Morina lehmanniana [M*. *coulteriana]*, *Festuca valesiaca*. On slopes with snow accumulations associated with meadow elements (*Polygonum bistorta [Bistorta major*, *Bistorta officinalis]*, *Allium polyphyllum [A*. *carolinianum]*, *Oxyria digyna*, *Pedicularis rhinanthoides*, *Primula turkestanica*). In the Jazgulom and Shugnan area (Figs 6 and 10) they are of secondary origin. In the Roshtkala area (Fig 8) also associated with *Artemisia lehmanniana*, *Trigonella griffithii [Melilotoides gontscharovii]*, *Carex duriusculiformis [C*. *stenophylla* subsp. *stenophylloides]*. In this area between 3,900 and 4,200 m asl a similar formation with *Poa relaxa* and *Xylanthemum pamiricum*, but nearly without *Acantholimon* species occurs.

In the moister Jazgulom area (Fig 6) steppes composed of different herbs are most important. Dominated by *Nepeta podostachys*, *Ziziphora bungeana [Z*. *clinopodioides]*, *Cousinia rubiginosa*, *Ephedra gerardiana* associated with steppe grasses (*Stipa caucasica*, *Zerna turkestanica [Bromopsis turkestanica*, *Bromus paulsenii]*, *Piptatherum platyanthum*, *Poa relaxa*) and herbs (*Erigeron amorphoglossus [Psychrogeton amorphoglossum]*, *Allium polyphyllum [A*. *carolinianum]*, *Chrysanthemum stoliczkae [Tanacetum stoliczkae]*, *Hypericum scabrum*, *Silene guntensis*). In high elevations it forms the ecotone to the cryophytic vegetation.

Odourous herbaceous steppes with *Nepeta podostachys*, *Ziziphora bungeana [Z*. *clinopodioides]*, *Origanum tyttanthum [O*. *vulgare* subsp. *gracile]*, *Dracocephalum paulsenii* associated with steppe grasses (e.g. *Stipa*, *Festuca*, *Zerna [Bromus]*, *Poa* species), mountain xerophytes (prickly *Acantholimon* species, *A*. *diapensioides*, *Ephedra*, *Arenaria [Eremogone]*), and on snow accumulations meadows herbs (*Geranium regelii*, *Polygonum coriarium [Aconogonon coriarium]*, *P*. *schugnanicum [Bistorta elliptica]*).

#### Prickly herbaceous steppes

Two different types: Typical *Cousinia pannosa* steppes associated with *Artemisia lehmanniania*, *A*. *persica*, *Ephedra fedtschenkoae*, *E*. *gerardiana*, *Astragalus roschanicus*, *Arenaria griffithii [Eremogone griffithii]*, *Ziziphora bungeana [Z*. *clinopodioides]*, *Acantholimon alatavicum* and sometimes steppe grasses (e.g. *Festuca valesiaca*, *Puccinellia subspicata*).

*Cousinia pannosa—C*. *stephanophora* steppes associated with *Artemisia persica* and meadow elements (*Kobresia* species, *Poa bucharica*, *P*. *zaprjagajevii*, *Polygonum coriarium [Aconogonon coriarium]*, *Dactylis glomerata*, *Geranium wachanicum [G*. *collinum]*) or with steppe grasses. Rushan area (Fig 7) near snow accumulations up to 3,700 m asl.

#### Wormwood steppes

Dominated by *Artemisia lehmanniana* associated with *Stipa orientalis*, *Acantholimon alatavicum*, *Cousinia rubiginosa*, *Arenaria griffithii [Eremogone griffithii]*, *Piptatherum platyanthum*, *Carex duriusculiformis [C*. *stenophylla* subsp. *stenophylloides]*, *Ephedra gerardiana*, *Ziziphora bungeana [Z*. *clinopodioides]*, *Festuca valesiaca*, *Onobrychis echidna*, *Allium jaquemontii [A*. *pamiricum]*.

### Tall forb communities

With 3 km^2^ (<0.1%), tall forb communities cover only very small parts of the study area. Their altitudinal distribution on the mapped area ranges between 2,178 and 3,930 (mean 2,754, median 2,631), the map legends list 3,900 m asl as the maximum elevation, and Agachanjanc [31] states a range between 3,100 and 3,300 m asl. The response curves shows a maximum probability of occurrence up to 3,000 m asl, followed by a decrease that reaches zero probability at 4,500 m asl.

#### *Prangos* vegetation

*Prangos pabularia* associated with *Polygonum coriarium [Aconogonon coriarium]*, *Heracleum olgae [Tetrataenium olgae]*, *Artemisia* species, steppe grasses. Sometimes associated with *Rosa* species, *Sorbus turkestanica*, *Cotoneaster multiflorus*. Shugnan area (Fig 10) on screes and alluvial fans.

### Mountain meadows

Mountain meadows occur in 12.8 km^2^ (0.3%) of the mapped area in elevations from 2,079 to 4,220 m asl (mean 3,276; median 3,335), with a flat probability peak between 3,000 and 3,600 m asl. In the map legend an altitudinal distribution between 1,700 and 4,100 m asl and in Agachanjanc [31] between 4,000 and 4,500 m asl is given. The rather big variability of this vegetation type and the discrepancy between the different data sources should be verified by future field work.

#### Knotweed meadows

**Fig 13:**
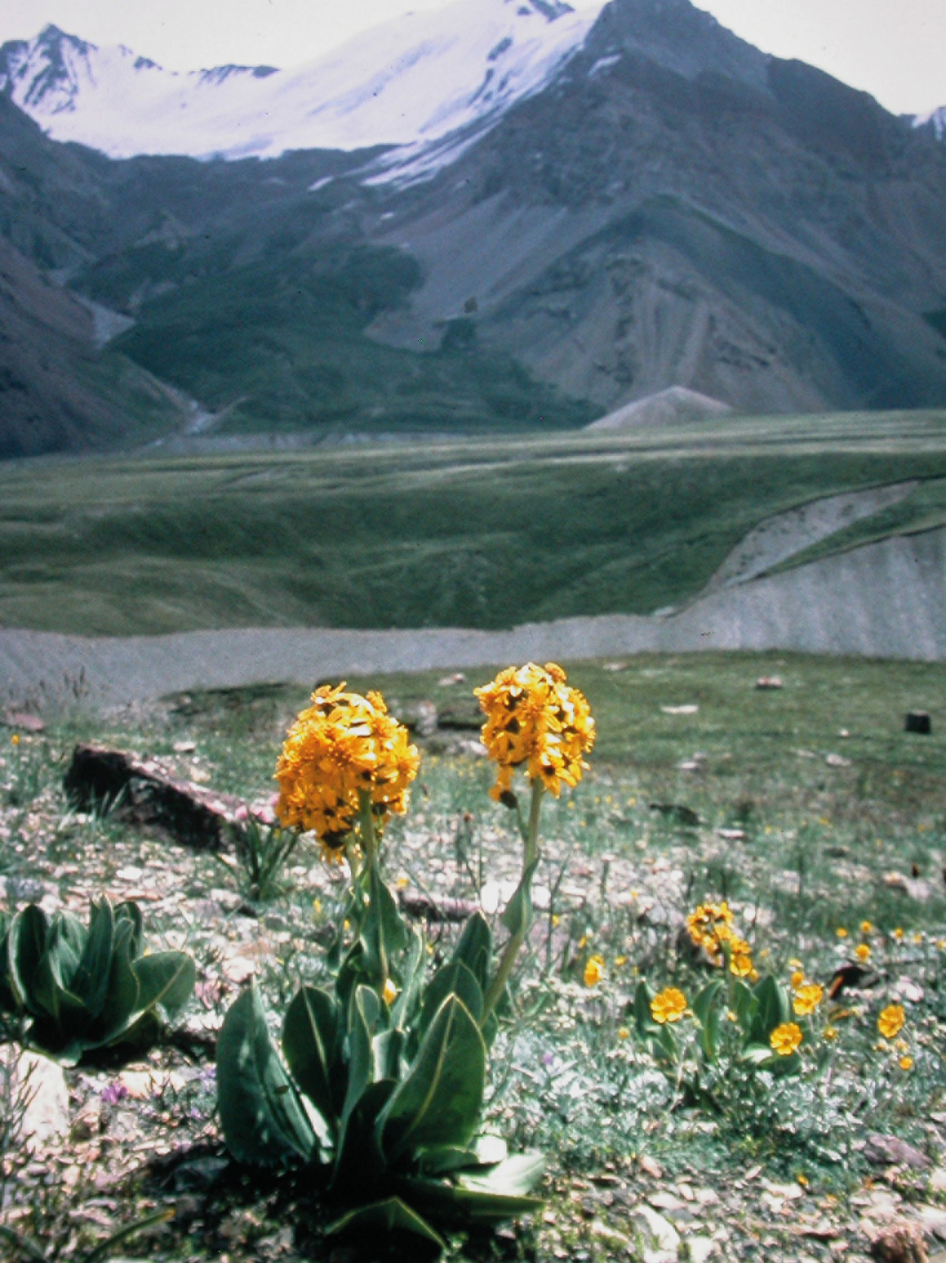
*Ligularia thomsonii*, a typical plant of knotweed meadows. Transalai Range, Murghab district, 4,200 m asl. (Photo: Agakhanjanz, 1976).

These meadows are dominated by the genus *Polygonum [Aconogonon]*. Several different types can be found throughout all mapped areas: *Polygonum coriarium [Aconogonon coriarium]* meadows associated with *Ligularia thomsonii* (Fig 13), *Vicia tenuifolia*, *Hordeum bulbosum*, *Dactylis glomerata*, *Prangos pabularia*, *Eremurus* species, *Cousinia stephanophora*, other high herbs and grasses. Jazgulom area (Fig 6) on slopes or alluvial fans.

*Polygonum bucharicum [Aconogonon bucharicum]* meadows. Pure or associated with *Chamaenerion angustifolium [Epilobium angustifolium]*, *Ziziphora bungeana [Z*. *clinopodioides]*, *Hypericum perforatum*, *Arenaria griffithii [Eremogone griffithii]*, *Ephedra gerardiana*, *Ferula jaeschkeana [F*. *kuhistanica]*, *Perovskia scrophulariifolia*, *Lindelofia macrostyla [L*. *anchusoides]*, *Roegneria interrupta [Elymus praeruptus]*. Jazgulom area (Fig 6) on alluvial fans or near snow accumulations.

Pure knotweed meadows (called *Taran*) composed of *Polygonum coriarium [Aconogonon coriarium]*. Infrequently associated with *Prangos pabularia*, *Ferula jaeschkeana [F*. *kuhistanica]*, *F*. *grigoriewii*, *Heracleum olgae [Tetrataenium olgae]*, *Rheum maximowiczii*, *Artemisia* species, *Ephedra tibetica [E*. *intermedia]*, *E*. *gerardiana*, *E*. *ciliata [E*. *kokanica]*. On alluvial fans and near snow accumulations.

Open *Polygonum coriarium* meadows associated with *Prangos pabularia*. On screes in the Shugnan area (Fig 10).

*Polygonum coriarium [Aconogonon coriarium]* meadows associated with shrubs (*Ribes janzcewskii*, *Rosa fedtschenkoana*), herbs (*Artemisia lehmanniana*, *Prangos pabularia*, *Ferula grigoriewii*, *Heracleum olgae [Tetrataenium olgae]*), and grasses (*Calamagrostis* species, *Stipa caucasica*, *S*. *badachschanica*). Roshtkala area (Fig 8) around springs.

### Floodplain meadows

Floodplain meadows are widespread in all mapped areas (94.0 km^2^, 1.8%) in elevations between 1,729 and 4,616 m asl (mean 3,695, median 3,667). In the map legends values between 2,700 and 4,000 m asl (sometimes 4,800 m asl) are given. The response curve shows a distinct probability peak of 0.85 at 3,700 m asl. These meadows are limited to riparian habitats under influence of groundwater or melting snow. Several associations can be differentiated, whereof the most important are dominated by sedges (*Carex*) and bog sedges (*Kobresia*) and show, according to field observations by the authors, degraded conditions due to a strong grazing impact.

#### Sedge meadows

**Fig 14:**
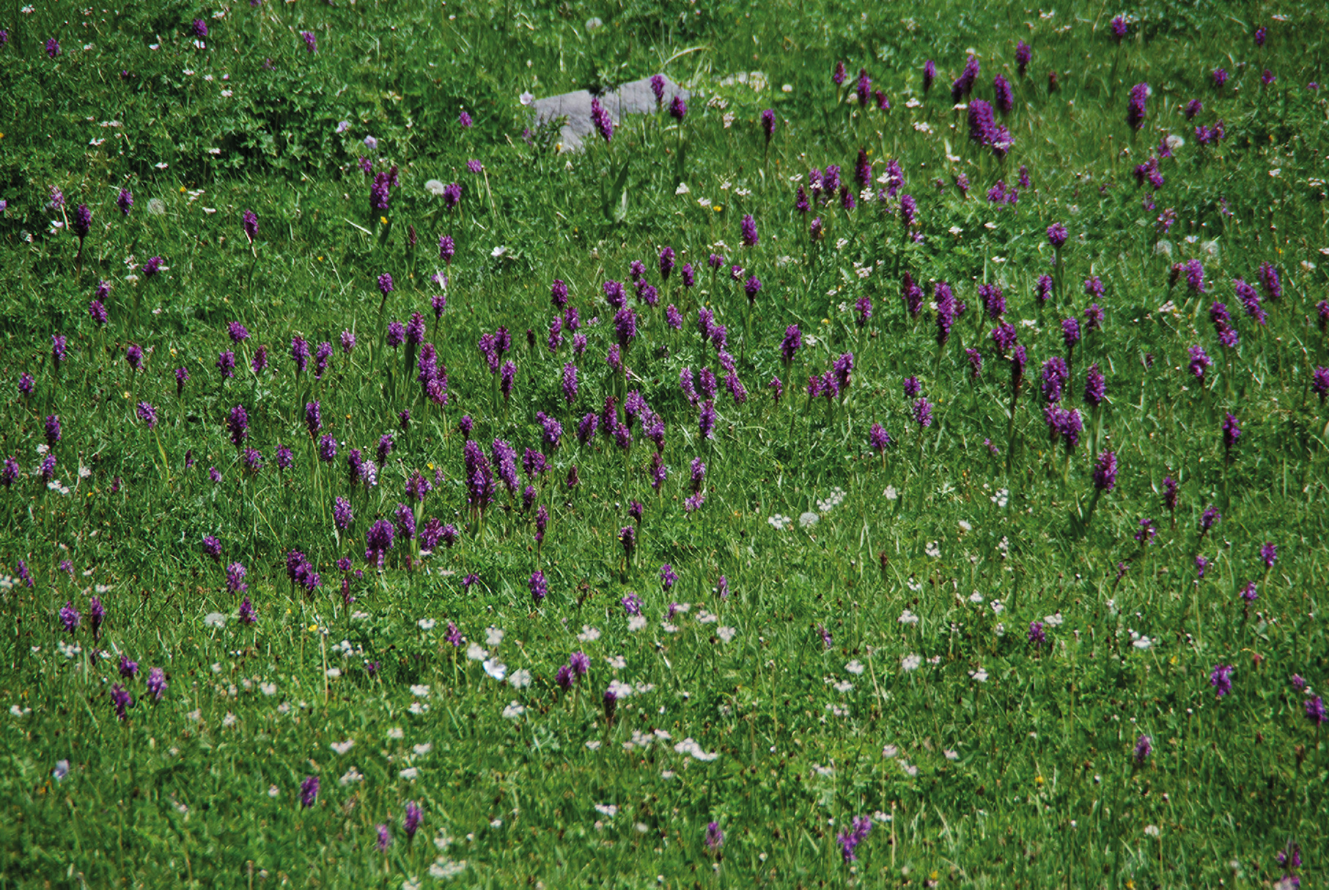
Sedge meadow with *Dactylorhiza umbrosa*. Badomdara Valley, Roshtkala district, 3,150 m asl. (Photo: Vanselow, 24.6.2014).

Dominated by *Carex griffithii [C*. *nivalis]* and *C*. *orbicularis* asscociated with *Blysmus compressus*, *Primula algida*, *P*. *warshenewskiana*, *Kobresia humilis*, *K*. *pamiroalaica*, *Alopecurus himalaicus*, *Bunium persicum*, *Scaligeria allioides [Elaeosticta allioides]*, *Poa pratensis*, *Ranunculus alajensis*, *Juncus macrantherus*, *Euphrasia tatarica [E*. *pectinata]*. Sometimes associated with *Calamagrostis dubia [C*. *pseudophragmites]*, *Myricaria alopecuroides [M*. *bracteata]*, *Lathyrus pratensis*, *Paracynoglossum denticulatum [Cynoglossum glochiadiatum]*, *Orchis umbrosa [Dactylorhiza umbrosa]* (Fig 14), *Trifolium* species. In swampy areas also associated with *Scirpus alpinus [Baeothyron alpinum]* and *Veronica beccabunga*. On overgrazed sites furthermore associated with *Swertia lactea*.

#### Bog sedge meadows

Dominated by *Kobresia humilis*, *K*. *pamiroalaica*, *and K*. *schoenoides* associated with many sedge species (e.g. *Carex orbicularis*, *C*. *pseudofoetida*, *C*. *melanantha*), *Trifolium repens [Amoria repens]*, *Agrostis alba [A*. *gigantea]*, *Ranunculus nivalis*, *Puccinellia hackeliana*, *P*. *akbaitalensis [P*. *pamirica]*, *Allium polyphyllum [A*. *carolinianum]*, *A*. *fedtschenkoanum*, *Alopecurus himalaicus*, *Saxifraga* spp., *Potentilla* spp.. On swamps also associated with *Triglochin palustris*.

### Cryophytic and subnival vegetation

Cryophytic and subnival vegetation covers 696.9 km^2^ (13.4%) of the study area. According to the data analysis this vegetation unit occurs between 2,748 (in a deeply incised north-exposed valley) and 5,254 m asl (mean 4,272, median 4,278), with a flat probability peak of nearly 1.0 between 3,700 and 5,000 m asl. Agachanjanc [31] states an altitudinal range between 4,000 and 5,000 m asl, the map legends give 4,800 m asl as the maximum value.

#### Cryophytic vegetation

**Fig 15:**
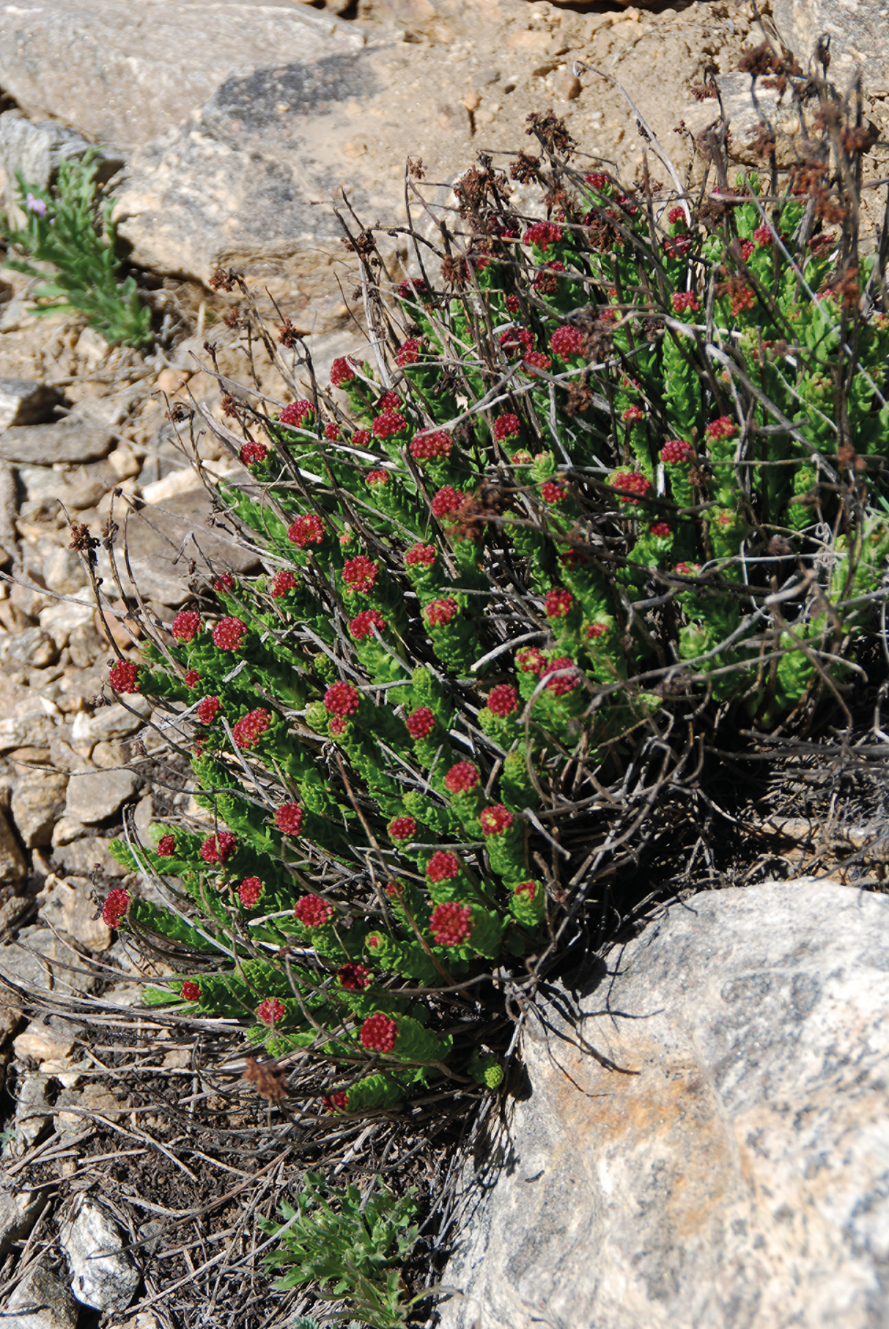
Sprouting male *Rhodiola heterodonta*, a typical cryophytic species in the Pamirs. Badomdara Valley, Roshtkala district, 4,060 m asl. (Photo: Vanselow, 25.6.2014).

No typical associations but disjunct low growing open aggregations that can be composed of several species, such as *Oxytropis immersa*, *O*. *savellanica*, *O*. *lapponica*, *Potentilla hololeuca*, *P*. *grisea*, *P*. *gelida*, *Draba korshinskyi*, *D*. *olgae*, *Smelowskia calycina*, *Lagotis ikonnikovii*, *L*. *cashmeriana*, *Ranunculus alajensis*, *Parrya exscapa [Leiospora exscapa]*, *Leontopodium ochroleucum*, *Arabis kokanica*, *Primula sibirica [P*. *nutans]*, *Myosotis alpestris*, *Hymenolaena pimpinellifolia [Pleurospermum stellatum* var. *lindleyanum]*, *Roegneria jacquemontii [Elymus longe-aristatus* subsp. *canaliculatus*, *Elymus jacquemontii]*, *Waldheimia tridactylites*, *Alopecurus mucronatus*, *Rhodiola heterodonta* (Fig 15), *Torularia korolkowii [Neotorularia korolkowii]*, *Allium fedtschenkoanum*, *Erigeron amorphoglossus [Psychrogeton amorphoglossum]*, *Corydalis gortschakovii*, *Hedysarum cephalotes [H*. *minjanense]*, *Atropis subspicata [Puccinellia subspicata]*, *Scutellaria* species, and others. In some places it shows steppe character (with *Puccinella subspicata* and *Allium polyphyllum [A*. *carolinianum]*) or it forms swamps and meadows with *Kobresia humilis*, *K*. *pamiroalaica*, *Carex orbicularis*, *Geranium regelii*, *Trisetum spicatum*, and *Allium fedtschenkoanum*.

#### Subnival vegetation

**Fig 16:**
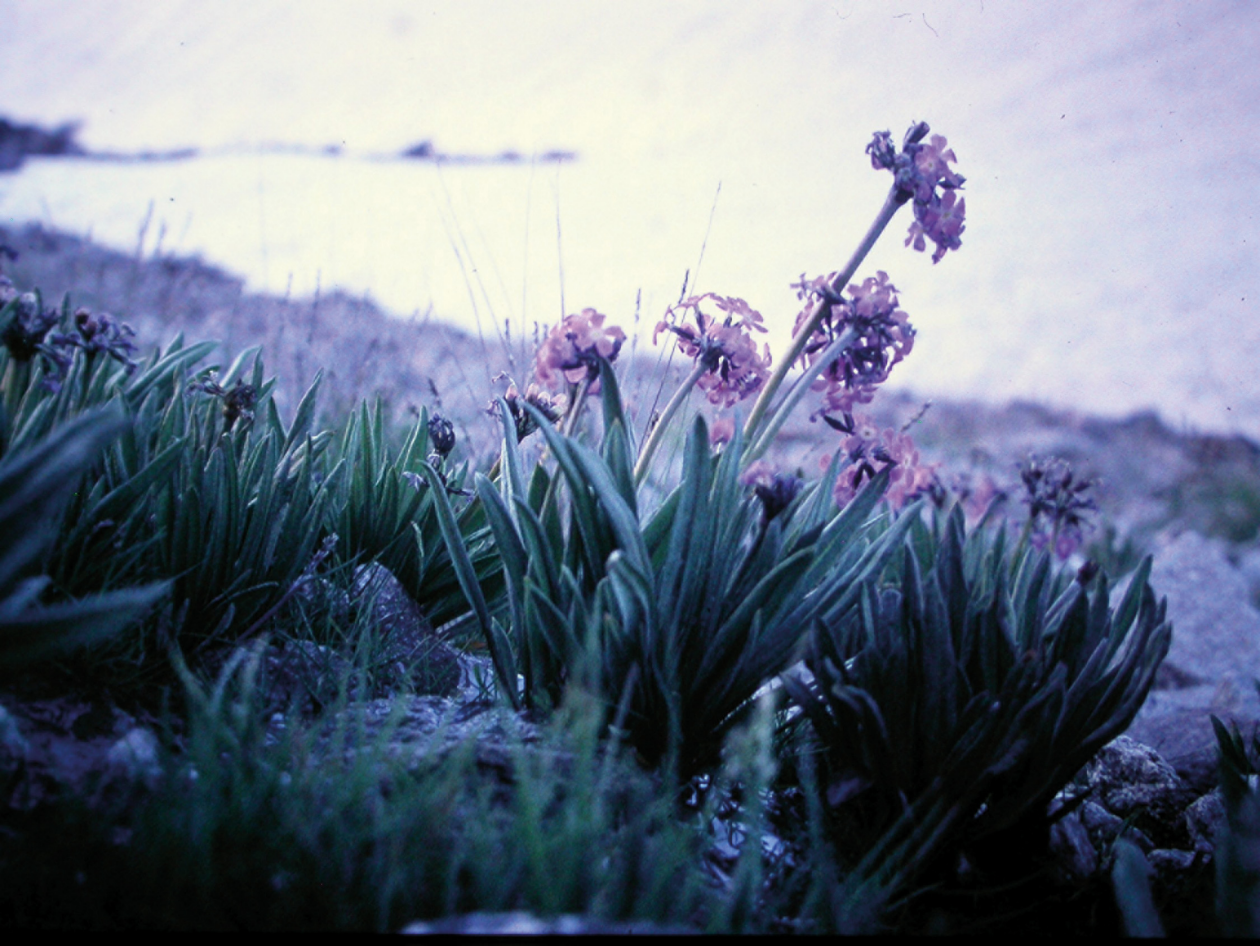
*Primula nivalis*. North of Turumtai Kul, Shugnan district, 5,000 m asl. (Photo: Breckle, 2002).

Important species of this vegetation type are *Primula sibirica [P*. *nutans]*, *P*. *ikonnikovii*, *P*. *nivalis* (Fig 16), *Oxytropis immersa*, *Lagotis ikonnikovii*, *Ranunculus nivalis*, *Sibbaldia olgae*, *Waldheimia tridactylites*, *Ermania incana [Oreoblastus incanus]*, *Hymenolaena pimpinellifolia [Pleurospermum stellatum* var. *lindleyanum]*, and *Parrya exscapa [Leiospora exscapa]*. On periglacial screes. Agakhanjanz does not mention the genus *Draba*, which is also a common element of the subnival vegetation (personal observation by the authors).

### Barren land

Barren land has the largest extent in the mapped area (2,705 km^2^, 52.1%) and occurs between 1,554 and 5,544 m asl (mean 3,874, median 4,018). The response curve displays a nearly linear increase of occurrence probability from 0.4 at 1,600 m asl to 0.6 at 6,000 m asl. While Agakhanjanz recorded predominantly very scattered vegetation in theses areas, preliminary remote sensing time-series analyses suggest a greening trend for this land cover type, which might be linked to increasing temperatures over the last 55 years.

#### Rocks

Rocks without vegetation or with single pioneer plants and lichens. Sometimes with single Juniper shrubs. Sometimes isolated stands with a special microclimate and by chance with single plants.

#### Screes

Screes, alluvial fans, and conglomerate slopes. With very scattered plant cover mostly composed of *Juniperus*, *Polygonum [Aconogonon]*, *Heracleum*, and *Prangos* species as well as steppe grasses.

Scattered *Ampelopsis vitifolia [A*. *aegirophylla]* patches. Jazgulom area (Fig 6).

Scree vegetation composed of *Heracleum olgae [Tetrataenium olgae]*, *Lactuca orientalis [Scariola orientalis]*, *Delphinium barbatum [Aconitella barbata]*, *Stipa badachschanica*, *Ceratoides papposa [Krascheninnikovia ceratoides]*, *Artemisia vachanica*. Jazgulom area (Fig 6).

Scree vegetation composed of *Prangos pabularia*, *Polygonum bucharicum [Aconogonon bucharicum]*, *Perovskia scrophulariifolia*, *Helichrysum mussae*, *Lonicera olgae*, *Rosa* species. Jazgulom area (Fig 6) in very high elevations.

Scree vegetation composed of *Prangos pabularia*, *Ferula grigoriewii*, *Heracleum lehmannianum*, *H*. *olgae [Tetrataenium olgae]*, *Polygonum coriarium [Aconogonon coriarium]*, *Rheum maximowiczii*, *Ephedra* spp., *Artemisia* spp.. On steep slopes only single plants of *Ampelopsis vitifolia [Amelopsis aegirophylla]*, *Perovskia scrophulariifolia*, *Cicer songaricum*, *Onobrychis echidna*, and sometimes *Acantholimon pamiricum*. Shugnan area (Fig 10).

In Roshtkala (Fig 8) most screes are without vegetation, depending on mobility of substrate. Rarely scattered associations with isolated *Prangos pabularia*, *Polygonum coriarium [Aconogonon coriarium]*, *Heracleum lehmannianum*, *H*. *olgae [Tetrataenium olgae]* and desert species occur. On alluvial fans sometimes *Rosa fedtschenkoana* and *Juniperus schugnanica [J*. *semiglobosa]*.

#### Moraines

Generally without vegetation cover.

#### Flood areas

Generally stony areas of the high mountain floodplains. Rarely single plants can occur (e.g. *Oxytropis michelsonii*, *Chrysanthemum stoliczkae [Tanacetum stoliczkae]*, *Roegneria ugamica [Elymus dentatus*, *E*. *nevskii]*).

### Nival areas

Nival areas consist of glaciers and firn fields without higher plants and cover 394.3 km^2^ (7.6%) of the mapped area. According to the response curves the probability for the occurrence is between 3,069 and 6,029 m asl (mean 4,679, median 4,673), while according to the map legends they are found above 4,600–4,800 m asl and above 3,900 m asl in the moister north (Jazgulom area). Agachanjanc [31] gives 4,500 m asl as the threshold for the occurrence of nival areas. This value is also displayed by the response curve that increases above 3,000 m asl and reaches a probability of 1.0 at around 4,500 m asl. Furthermore, for this land cover class a relatively high relevance of north-exposedness could be verified by the data analysis. The response curve shows lowest probability values for values below zero (i.e. south-exposedness) and a linear increase of probability from 0 to 1.0 (i.e. fully north-exposed). Compared to the 1960s the extension of nival areas decreased due to strong warming in this region [17].

### Cultivated land

**Fig 17:**
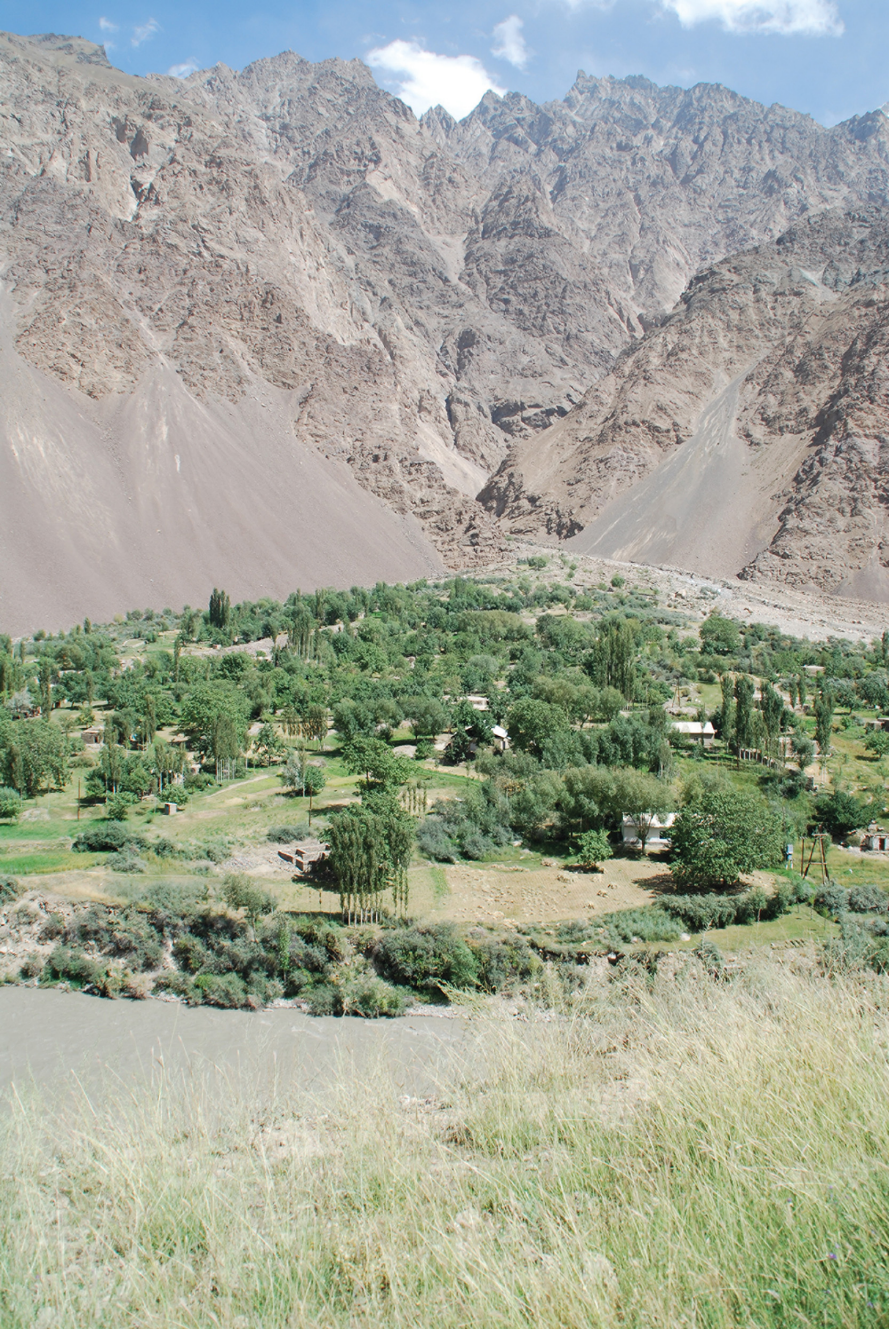
Cultivated land on an alluvial fan. Gunt valley, Shugnan district, ca. 2,400 m asl. (Photo: Vanselow, 28.8.2011).

This land cover class predominantly consists of settlements, agricultural land, clover meadows, gardens (see Fig 17), and in the Jazgulom area (Fig 6) also of walnut plantations with *Juglans regia*. Cultivated land covers 133.5 km^2^ (2.6%) of the mapped area in elevations between 1,596 and 3,930 m asl (mean 2,560, median 2,570). The response curve indicates a maximum probability of occurrence up to 3,800 m asl, followed by a sharp drop towards zero probability. According to the map legends cultivated land reaches up to 3,400 m asl (rarely 3,700 m asl). For this land cover class also slope was identified as an important environmental variable. The response curve shows a nearly linear trend of a maximum probability at 0° via a 0.5 probability at just under 30° towards zero probability at 70°.

## Conclusion

The presented maps depict a detailed description of the distribution and the status of vegetation in the study area in 1958–1960. However, they also have some weaknesses that need to be outlined. Ground checks revealed spatial inaccuracies of the polygons. This applies particularly to highly elevated polygons, e.g. of cryophytic and subnival vegetation, and to very narrow polygons depicting Mountain Tugai, floodplain meadows and cultivated land. For example, edges of narrow Mountain Tugai polygons reached into the valley slope and hence into neighbouring vegetation classes like e.g. *Artemisia* deserts. Another problem is the unclear botanical taxonomy. Many species names are outdated or need to be verified and existing determinations are often doubtful or highly debated by taxonomists. In addition, the number of species listed in the map descriptions is far from being complete. The estimated number of species for the entire Western Pamirian flora is between 1,500 [2] and 2,000 [3]. Obviously, in the map descriptions only prominent and/or dominant species are listed. This is mainly due to the fact that mapping at that time had the main goal to provide data on productivity of the various vegetation types. Therefore, further taxonomic efforts are necessary, including the collection of herbarium specimen. Nevertheless, we are confident that the maps presented in this article provide a sound basis for the study of environmental changes that e.g. occurred widespread after the breakdown of the Soviet Union, and recently because of increasing temperatures and heavy rains that can lead to extreme events, such as floods, debris flows, or glacial lake outbursts [5,42,43]. For example, on the 7^th^ August 2002 a glacial lake outburst destroyed the village of Dasht in the Shakhdara valley, killed 24 people and displaced the Shakhadar river bed by about 1 km. A similar event occurred in the Red valley (a tributary valley of the Bartang valley in the Rushan area) where heavy rains caused a debris flow in summer 2011 that destroyed the cultivated land and Tugai forests almost completely causing the abandonment of three small villages. Finally, since vascular plants react with some delay on changed environmental conditions, they are considered to display long term trends and thus are useful indicators for an ecological assessment of the impact of climate change [23,25]. Particularly, high mountain areas are ideal sites for comparative studies of cold habitats [25] and the maps presented here can serve as a comparison baseline.

## References

[1] Beg GA (2009) Cross-Border Cooperation for Biodiversity Conservation and Sustainable Development. Case Studies on Karakoram, Hindukush and Pamir In: Kreutzmann H, Beg GA, Richter J, editors. Experiences with and Prospects for Regional Exchange and Cooperation in Mountain Areas. Kathmandu: InWEnt pp. 184–211.

[2] Breckle S-W, Wucherer W (2006) Vegetation of the Pamir (Tajikistan): Land Use and Desertification Problems In: Spehn E, Liberman M, Körner C, editors. Land-use Change and Mountain Biodiversity. Boca Raton: CRC/Taylor & Francis pp. 225–237.

[3] Agakhanjanz OE, Breckle S-W (1995) Origin and evolution of the mountain flora in Middle Asia and neighboring mountain regions. Ecological Studies 113: 63–80.

[4] Akhmadov K, Breckle S-W, Breckle U (2006) Effects of Grazing on Biodiversity, Productivity, and Soil Erosion of Alpine Pastures in Tajik Mountains In: Spehn E, Liberman M, Körner C, editors. Land Use Change and Mountain Biodiversity. Boca Raton: CRC/Taylor & Francis pp. 239–247.

[5] Kassam KA (2009) Viewing change through the prism of indigenous human ecology: Findings from the afghan and Tajik pamirs. Human Ecology 37: 677–690.

[6] Giuliani A, Van Oudenhoven F, Mubalieva S (2011) Agricultural biodiversity in the Tajik Pamirs. Mountain Research and Development 31: 16–26.

[7] Salick J, Zhendong F, Byg A (2009) Eastern Himalayan alpine plant ecology, Tibetan ethnobotany, and climate change. Global Environmental Change 19: 147–155.

[8] Breu T, Hurni H (2003) The Tajik Pamirs Challenges of Sustainable Development in an Isolated Mountain Region. Berne: Centre for Development and Environment (CDE).

[9] Qonunov Y (2010) Recent Changes in Pastoral Systems. Case Study on Tajikistan In: Kreutzmann H, Abdulalishoev K, Zhaohui L, Richter J, editors. Pastoralism and Rangeland Management in Mountain Areas in the Context of Climate and Global Change. Khorog, Kashgar: giz, BMZ pp. 82–101.

[10] Mislimshoeva B, Samimi C, Kirchhoff JF, Koellner T (2013) Analysis of costs and people's willingness to enroll in forest rehabilitation in Gorno Badakhshan, Tajikistan. Forest Policy and Economics 37: 75–83.

[11] Kayumov A, Rajabov I (2010) Glaciers–Water Resources of Tajikistan in Condition of the Climate Change. Dushanbe: State Agency for Hydrometeorology of Committee for Environmental Protection under the Government of the Republic of Tajikistan.

[12] Khromova TE, Osipova GB, Tsvetkov DG, Dyurgerov MB, Barry RG (2006) Changes in glacier extent in the eastern Pamir, Central Asia, determined from historical data and ASTER imagery. Remote Sensing of Environment 102: 24–32.

[13] Oberhänsli H, Novotná K, Píšková A, Chabrillat S, Nourgaliev DK, et al (2011) Variability in precipitation, temperature and river runoff in W Central Asia during the past ~ 2000 yrs. Global and Planetary Change 76: 95–104.

[14] Narama C (2001) Glacier variations in the Pamir‐Alai and West Tien Shan mountains, Central Asia over the last ninety years. Geographical Reports of Tokyo Metropolitan University 36: 37–48.

[15] Chevallier P, Pouyaud B, Mojaïsky M, Bolgov M, Olsson O, et al (2014) River flow regime and snow cover of the Pamir Alay (Central Asia) in a changing climate. Hydrological Sciences Journal 59: 1491–1506.

[16] Ohara N, Jang SH, Kure S, Richard Chen ZQ, Kavvas ML (2014) Modeling of interannual snow and ice storage in high-altitude regions by dynamic equilibrium concept. Journal of Hydrologic Engineering 19: 04014034.

[17] Pachauri RK, Reisinger A, editors (2007) Climate Change 2007: Synthesis Report Contribution of Working Groups I, II and III to the Fourth Assessment Report of the Intergovernmental Panel on Climate Change. Geneva: IPCC.

[18] Herbers H (2006) Landreform und Existenzsicherung in Tadschikistan Die Handlungsmacht der Akteure im Kontext der postsowjetischen Transformation. Erlangen: University of Erlangen-Nuremberg.

[19] Frei E, Bodin J, Walther G-R (2010) Plant species’ range shifts in mountainous areas—all uphill from here? Botanica Helvetica 120: 117–128.

[20] Gottfried M, Pauli H, Futschik A, Akhalkatsi M, Barancok P, et al (2012) Continent-wide response of mountain vegetation to climate change. Nature Climate Change 2: 111–115.

[21] Mamatumarov R (2011) Les plantes medicinales du Pamir Oriental et leur utilisation en medecine populaire Bishkek: Foundation Kyrgyz Ate.

[22] Grabherr G, Gottfried M, Pauli H (1994) Climate effects on mountain plants. Nature 369: 448. 23320303 10.1038/369448a0

[23] Walther G-R, Beißner S, Burga CA (2005) Trends in the Upward Shift of Alpine Plants. Journal of Vegetation Science 16: 541–548.

[24] Löffler J, Anschlag K, Baker B, Finch OD, Diekkrüger B, et al (2011) Mountain ecosystem response to global change. Erdkunde 65: 189–213.

[25] Grabherr G, Pauli H, Gottfried M (2010) A worldwide observation of effects of climate change on mountain ecosystems In: Borsdorf A, Grabherr G, Heinrich K, Scott B, Stötter J, editors. Challenges for mountain regions—tackling complexity. Vienna: Böhlau Verlag pp. 48–57.

[26] Agachanjanz O (1980) Auf dem Pamir Aufzeichnungen eines Geobotanikers. Moskau: Verlag Progreß.

[27] Agachanjanz OE (2002) Der Wind, der heißt Afghane Forschungen auf dem Pamir im Jahr der Schlange. Aachen: Shaker.

[28] Böhner J (2006) General climatic controls and topoclimatic variations in Central and High Asia. Boreas 35: 279–295.

[29] Schiemann R, Lüthi D, Vidale PL, Schär C (2008) The precipitation climate of Central Asia—Intercomparison of observational and numerical data sources in a remote semiarid region. International Journal of Climatology 28: 295–314.

[30] Walter H, Breckle S-W (1994) Ökologie der Erde Bd. 3. Spezielle Ökologie der gemäßigten und arktischen Zonen Euro-Nordasiens Zonobiom VI—IX. Stuttgart: Fischer.

[31] Agachanjanc O (1985) Ein ökologischer Ansatz zur Höhenstufengliederung des Pamir-Alai. Petermanns Geographische Mitteilungen 129: 17–23.

[32] Miehe G, Winiger M, Böhner J, Yili Z (2001) The climatic diagram map of High Asia. Purpose and concepts. Erdkunde 55: 94–97.

[33] Stone PB (1992) The State of the World's Mountains A global report. London, New Jersey: Zed Books Ltd.

[34] USGS (2013) Shuttle Radar Topography Mission.

[35] Leyer I, Wesche K (2007) Multivariate Statistik in der Ökologie Eine Einführung. Berlin, Heidelberg: Springer.

[36] Hastie TJ, Tibshirani RJ (1990) Generalized Additive Models. New York: Chapman & Hall.

[37] McCullagh P, Nelder JA (1989) Generalized Linear Models. London: Chapman and Hall.

[38] Yee TW, Mitchell ND (1991) Generalized Additive Models in Plant Ecology. Journal of Vegetation Science 2: 587–602.

[39] Wood S (2014) Mixed GAM Computation Vehicle with GCV/AIC/REML smoothness estimation (mgcv). 1.8–4 ed.

[40] Czerepanov SK (1995) Vascular Plants of Russia and Adjacent States (the Former USSR). Cambridge: Cambridge University Press.

[41] Breckle S-W, Hedge IC, Rafiqpoor MD (2013) Vascular Plants of Afghanistan–an augmented Checklist Dittmann A, editor. Bonn, Manama, New York, Florianapólis: Scientia Bonnensis. 598 p.

[42] Mergili M, Schneider JF (2011) Regional-scale analysis of lake outburst hazards in the southwestern Pamir, Tajikistan, based on remote sensing and GIS. Natural Hazards and Earth System Science 11: 1447–1462.

[43] Gruber FE, Mergili M (2013) Regional-scale analysis of high-mountain multi-hazard and risk indicators in the Pamir (Tajikistan) with GRASS GIS. Natural Hazards and Earth System Sciences 13: 2779–2796.

